# The Pathogenicity Mechanisms of *Staphylococcus aureus*

**DOI:** 10.3390/ijms262411803

**Published:** 2025-12-06

**Authors:** Beata Mlynarczyk-Bonikowska, Lidia Rudnicka

**Affiliations:** Department of Dermatology, Medical University of Warsaw, 59 Nowogrodzka Street, 02-006 Warsaw, Poland; lidia.rudnicka@wum.edu.pl

**Keywords:** *Staphylococcus aureus*, pathogenicity, biofilm, regulation systems, toxins, superantigens, skin diseases

## Abstract

*Staphylococcus aureus* is one of the most important bacterial pathogens affecting both humans and animals. This review discusses the most significant factors that contribute to the pathogenicity of these bacteria and the mechanisms that regulate their expression. We also focus on the factors that play a role in the pathogenesis of skin diseases. *S. aureus* possesses a wide array of virulence factors that allow it to bypass passive and active mechanisms of the host’s immune system and effectively infect and spread within the infected organism. These include factors that facilitate colonization of the skin (i.e., arginine catabolic mobile element-ACME), mucous membranes and other surfaces, proteins protecting the bacteria from the host’s immune system, superantigens and superantigen-like proteins, surface proteins that promote adhesion and biofilm formation, toxins, enzymes, and iron uptake systems. Additionally, a complex network of regulatory systems (accessory gene regulator -Agr, (staphylococcal accessory regulator -Sar, *S. aureus* exoprotein expression -Sae, and others) controls the expression of virulence genes at the transcriptional and translational levels. The activity of these regulatory systems is pivotal in determining whether *S. aureus* initiates an invasive infection, characterized by toxin and enzyme production (e.g., hemolysin alpha -Hla, phenol soluble modulins -PSM, toxic shock syndrome -TSST-1, enterotoxins, Panton-Valentine leukotoxin- PVL). This is indicative of community-acquired *S. aureus* (CA-Sa, CA-MRSA, CA-MSSA), or a chronic infection, characterized by surface protein expression and biofilm formation, which is indicative of hospital-acquired or healthcare-acquired *S. aureus* (HA-Sa, HA-MRSA, HA-MSSA).

## 1. Introduction

*Staphylococcus aureus* is one of the most significant bacterial pathogens affecting humans and animals. It is the most common cause of skin and soft tissue infections, but can also cause deeper or systemic infections, including toxic shock syndrome, pneumonia, sepsis, endocarditis, and osteomyelitis. Due to their sensitivity to beta-lactam antibiotics resistant to beta-lactamases, *S. aureus* strains are divided into MRSA (methicillin-resistant *S. aureus)*, which is resistant to all beta-lactam antibiotics except ceftobiprole and ceftaroline), and MSSA (methicillin-sensitive *S. aureus*). MSSA is sensitive to isoxazolyl penicillins and cephalosporins but may be resistant to benzylpenicillin and ampicillin if the strain produces beta-lactamase.

*S. aureus* strains are also categorized by epidemiology as CA-Sa (community-acquired *S. aureus*), CA-MRSA or CA-MSSA [1], HA-MRSA or HA-MSSA (hospital-acquired or healthcare-associated MRSA or MSSA) [2], or LA-MRSA or LA-MSSA (livestock-associated MRSA or MSSA) [3].

The epidemiological characteristics of *S. aureus* strains were analyzed using whole genomic DNA, which was first digested with SmaI, then with EagI and SacII endonucleases, before being separated using the PFGE (Pulsed-Field Gel Electrophoresis) method. This method was used to isolate clones such as USA300 and others [4]. Subsequent studies were based on whole genome sequencing (WGS) and included determining: the MLST (multilocus sequence typing) and clonal complex (CC) types, the SCCmec cassette type and subtype (staphylococcal chromosomal cassette mec types I–XIV) in MRSA, and the Spa (a polymorphic VNTR in the 3′ coding region of the *S. aureus*-specific staphylococcal protein A) type. The *agr* (accessory gene regulator) type (1, 2, 3, or 4), the presence of Panton-Valentine leukotoxin genes (PVL) or prophages encoding leukotoxins, the ACME (arginine catabolic mobile element), and the pathogenic island (SaPI) are also commonly determined [5,6]. Identifying epidemiological types of *S. aureus* and their molecular characteristics makes it possible to track their spread on a global and local scale and to implement proven methods of treatment and prevention.

The pathogenicity of *S. aureus* is associated with the presence of many different virulence factors encoded by several dozen genes. Some of these genes are present in most strains (e.g., the alpha toxin gene), while others are characteristic of certain groups (e.g., leukotoxin genes are much more common in CA-Sa). The mere presence of genes does not guarantee their expression. The pool of virulence factors produced by the *S. aureus* strain depends on the activity of many regulatory systems. Gene expression is regulated at two levels: transcription (e.g., activation or inhibition of the repressor gene, dissociation of the repressor protein, or inhibition of the gene promoter) and translation. Many gene transcripts (e.g., the *hla-* hemolysin alpha gene transcript) have an RBS (ribosome binding site) that is inaccessible to the ribosome. Induction at the translation level involves interaction between the gene transcript and the regulatory system transcript. In the case of the *hla* gene transcript, for example, it binds to the homologous 5′ end of the *agr*RNAIII transcript, making the RBS of the *hla* transcript available and allowing translation to occur. Transcripts of regulatory systems can be coding (mRNA) or non-coding (sRNA). The same system can produce different transcripts with different properties from different promoters. These transcripts can, as a result of interacting with another transcript, induce the translation of some genes and inhibit the translation of others. *S. aureus* has many regulatory systems for both levels of regulation [7,8].

When assessing the pathogenicity of individual *S. aureus* groups, CA-SA strains appear to have the greatest pathogenic potential, especially CA-MRSA. These strains have a larger pool of pathogenicity genes than others. They produce LukFS-PV (Panton–Valentine leukocidin) and other leukotoxins much more frequently, and we detect pathogenicity islands containing superantigen genes much more frequently in them. These include, for example, SaPI1, SaPI2 (e.g., st. SR434; ST88), SaPI3 (e.g., st. FR3757; ST8), SaPI4 (e.g., st. MW2; ST1), Sa alpha (e.g., st. SR434; ST88), Sa beta (contains *lukDE* leukotoxin genes, e.g., st. SR434; ST88), Sa gamma (e.g., st. SR434; ST88), SaPIsaitama (contains *tst*, *sec*, *sel* genes; ST834) incorporated into the chromosome DNA of bacteriophages phi SA1, phi SA2 (contains PVL leukotoxin genes), e.g., (st. MW2; ST1); e.g., (st. FR3757; ST8), phi SA3 (contains *chp*, *scn*, and *sak* genes), ACME element in USA300 NAE (North American lineage), e.g., (st. FR3757; ST8) enabling colonization of healthy skin, and COMER element (resistance to copper and mercury in USA300 SAE (South American line)) [9,10,11,12,13]. In Europe, the dominant CA-MRSA clone is CC80 (ST80/IV), but other clones also occur, e.g., USA300 [14,15]. The regulation of pathogenicity gene expression in CA-Sa strains (MRSA and MSSA) is usually associated with the Agr system, whose activity determines the expression of many toxins and enzymes, which may be the cause of invasive infections. However, CA-Sa strains have a much smaller pool of genes determining antibiotic resistance than HA-Sa strains. CA-MRSA strains most often carry SCCmec type IV, which determines resistance to most beta-lactam antibiotics. HA-Sa strains most often have an inactive Agr regulatory system, which determines the lack of expression of many toxins, the expression of surface proteins, and the formation of biofilms, leading to chronic infections. Despite their higher virulence potential, severe CA-MRSA infections (bacteremia, pneumonia) usually have a lower mortality rate (15–30%) compared to similar HA-MRSA infections (30–50%), which is explained by differences in the existence of comorbidities in hospitalized patients and the fact that the appropriate antibiotic is not always used in the initial period of treatment, before the bacteriological result is obtained.

Within *S. aureus*, aside from the biotype that is responsible for human disease, there are also animal biotypes (typical of horses, sheep, cattle, and others) that are not generally considered to be pathogenic to humans. From this group of zoonotic *S. aureus* in areas with high livestock density, a group of LA-Sa (LA-MRSA, LA-MSSA), mainly ST398, has emerged, which can cause infections in humans. LA-Sa has a relatively small number of pathogenicity and antibiotic resistance factors. The active Agr system in these strains stimulates the production of alpha toxin and many other regulatory systems, e.g., ESAT-6 (ESS), PhoP, SrrB, WalK, SarX, SigB, and ClpP. So far, only a few superantigens, such as enterotoxins SEA SEP encoded by phiSa3 prophage and SElW (staphylococcal enterotoxin like W), have been described. The EsxX protein specific to the Chinese lineage ST398, which determines neutrophil lysis and resistance to killing by neutrophils, has also been described. LA-MRSA/LA-MSSA strains (ST398 and ST9), especially those that synthesize enterotoxins, may pose a threat in agricultural regions. LA-MRSA infection may result from cross-contamination, contact with raw meat, or consumption of undercooked meat. LA-SA strains that infect humans usually contain phiSa3 [11].

## 2. The Factors Responsible for the Colonization of Mucous Membranes and Healthy Skin During the Initial Stage of Infection

A key role in the colonization of mucous membranes is played by proteins that bind covalently to the nasal epithelium, such as ClfB (clumping factor B, fibrinogen and keratin-10 binding surface anchored protein, microbial surface components recognizing adhesive matrix molecules, MSCRAMM family protein) [16], SasG, SasX (*S. aureus* surface protein G or X) [17,18], Pls (plasmin sensitive protein) [19], IsdA (iron-regulated surface determinant protein A) [16], SdrC (MSCRAMM family adhesin, SEI serine-aspartate rich fibrinogen-binding, bone sialoprotein-binding protein), and SdrD (serine-aspartate repeat-containing protein D, sialoprotein-binding protein). SasG and ClfB also have the ability to bind to skin components in the epidermis and cause inflammation. The most important role in colonizing healthy skin is played by ACME (arginine catabolic mobile element) and SpeG protein (spermidine N-acetyltransferase). *S. aureus* easily colonizes mucous membranes (e.g., nasal epithelium), but colonization of healthy skin is difficult due to *S. aureus*’ high sensitivity to polyamines (spermine, spermidine) produced by the skin and its sensitivity to low skin pH. The situation changed when some CA-MRSA strains (e.g., USA300) acquired the mobile ACME and SpeG elements from *Staphylococcus epidermidis*, which should be considered an undoubted evolutionary success. The presence of ACME increases skin colonization by neutralizing the acidic pH of human sweat. This is achieved by the arginine deiminase system encoded in ACME through the production of ammonia from the catabolism of l-arginine. ACME also improves the survival and growth of *S. aureus* on human skin by depleting l-arginine, which is the only substrate to produce nitric oxide by macrophages producing the nitric oxide synthase isoform (iNOS). Spermidine N-acetyltransferase encoded by the *speG* gene counteracts polyamine toxicity [20,21,22]. ACME I contains the arginine deiminase pathway gene cluster (ACME-arc) and the oligopeptide transporter gene cluster (ACME-opp) [23]. The 6174 bp ACME element region contains the *argR*, *argC*, *argD*, *arcB*, and *arcC* genes, encoding, respectively: arginine repressor ArgR, arginine deiminase ArgC, arginine/ornithine antiporter ArgD, ornithine carbamoyltransferase ArcB, and carbamate kinase ArcC (GenBank acc. KC879557) [24]. ACME type I (ACME I) has been identified in PVL-positive (PVL encoded in prophage, phiSA2usa) ST8 MRSA with SCCmec type IVa (USA300 clone) [23], and also occurs in other epidemiological lineages at a low frequency. ACME type II′ (previously referred to as ACME ΔII) has been detected in MRSA isolates with SCCmec II, V, and XIV. ACME type II (ACME II) or its shortened variant ACME II′ (previously referred to as ΔACME II) includes ACME-arc but does not include ACME-*opp* or *speG*, which may be encoded in some strains by SCC mec. In studies on MRSA carriage in regions where ACME-positive strains are present, a swab is taken from the groin rather than from the nasal mucosa, which significantly increases the chances of culturing them [25,26].

## 3. Factors That Protect Bacteria from the Host’s Immune System

To survive within the host, *S. aureus* deploys a sophisticated arsenal of proteins that directly undermine the immune response. These can be categorized into several functional groups, including (1) complement-inhibiting proteins, such as Efb (extracellular fibrinogen binding protein, complement convertase inhibitor), Ecb (extracellular complement binding protein) [27], and Sbi (*S. aureus* binder of IgG) [16]; (2) proteins that prevent phagocytosis and killing by human neutrophils, such as Scn (SCIN,116 aa; staphylococcal complement inhibitor, a gene situated on a *hlb*-integrating phage phiSA3 or SaPIpig2) [28], eqSCIN (equid variant of staphylococcal complement inhibitor) in phage Saeq1, and Chp (CHIPS, chemotaxis-inhibiting protein situated on a prophage phiSA3 [29,30,31]; (3) immunoglobulin-binding proteins Sbi and Spa (*S. aureus* immunoglobulin G binding protein A); (4) superantigens and superantigen-like proteins; (5) biofilms; and (6) capsules.

### 3.1. Complement-Inhibiting Proteins

Three families of proteins (Efb, SCIN, and Sbi) have been described that bind to and impair the function of C3b through different mechanisms:

The Efb family includes Efb proteins (extracellular fibrinogen binding protein, complement convertase inhibitor). The only sequence in GenBank for the *efb* gene concerns *Staphylococcus schweitzeri* and Ecb (extracellular complement binding protein, a protein commonly found in *S. aureus*) [32]. Both proteins bind to the C3d part of C3b, thereby inhibiting the formation of new C3 convertases and enhancing the regulatory function of the host’s FH (plasma regulator factor H). The amino terminus of Efb binds to fibrinogen and impairs platelet function. Ecb does not have a fibrinogen-binding domain but binds to both FH and C3b to increase bacterial virulence [30].

The SCIN (staphylococcal complement inhibitor) family of proteins, SCIN, SCIN-B, and SCIN-C, stabilize and block the activity of C3 convertase. In addition, SCIN causes dimerization of C3 convertase. It has also been demonstrated that the binding of FH CR1 and the complement receptor of the Ig superfamily (CRIg) to such dimeric convertases is impaired [30].

A protein from the third family, Sbi, blocks the binding of Fcgamma receptors to IgG and forms ternary complexes with C3b and FH, causing a reduction in complement activation also binds to C3, leading to the activation of an alternative complement pathway and the consumption of this component, and stabilizes C3d dimers by inducing an N-terminal helix swap [30] (Table 1).

### 3.2. Proteins That Prevent Phagocytosis and Killing by Human Neutrophils

*S. aureus* strains originating from humans often contain the phiSa3 prophage, which converts the beta-hemolysin (*hlb*) gene and encodes the IEC (immune evasion cluster) genes *chp* and *scn*. The proteins encoded by these genes, CHIPS (neutrophil chemotaxis-inhibiting protein) and SCIN (staphylococcal complement inhibitor), allow *S. aureus* to evade phagocytosis and killing by human neutrophils [33]. In addition, IEC phiSa3 encodes the Sak (staphylokinase; defensin inhibitor), and superantigens-enterotoxins SEA and SEP (ST398), and is the primary virulence factor of these bacteria. It integrates with attB, with a core sequence (5′-TGTATCCAAACTGG-3′) in the *hlb* (hemolysin B) gene of the *S. aureus* chromosome. It is 42,700–43,000 bp in size, and the genome encodes 64–68 genes. The phiSa3 prophage is most common in CA-MRSA and is present in subclades of CC398 (LA-MRSA) of human origin. The IEC genes of the phiSa3 prophage play a key role in switching the host from animals to humans in LA-MRSA/MSSA strains [34].

### 3.3. Immunoglobulin-Binding Proteins

Spa is a protein anchored in the cell wall of *S. aureus*. Spa has two different Ig binding sites: Fab and Fc. The binding of Spa to the Fc region of IgG causes the bacterial cell to be coated with IgG, which cannot be recognized by Fc receptors on neutrophils and cannot catalyze complement binding. Bacteria deficient in or lacking protein A are more susceptible to capture and killing by neutrophils in the presence of serum opsonins [35,36,37,38].

Sbi is non-covalently bound to lipoteichoic acid in the cytoplasmic membrane of *S. aureus* cells and is not anchored in the cell wall. Sbi consists of two IgG-binding domains, similar to the domains of protein A, and a region that triggers the activation of complement C3. Sbi is expressed on the cell surface [16,35,36,37,39]. The most important proteins protecting *S. aureus* from the host’s immune system are presented in Table 2 according to previous studies [16,27,28,31,37,38,39,40,41,42].

### 3.4. Superantigens and Superantigen-like Proteins

This group includes the most important and well-known *S. aureus* toxins causing, among others, toxic shock syndrome (TSST-1) and food poisoning (enterotoxins). Certain aspects are described further in Chapter 6—Toxins. The term superantigen (SAg) was introduced in 1989 by White et al. [43]. It covers a group of bacterial proteins which, as unprocessed molecules, interact non-specifically with the variable part of the beta chain of the T-cell receptor (TCR) within the TCR-MHC class II complex. In humans, there are 56 different TCRVbeta, comprising 26 major classes of beta chains in the TCR [44]. SAgs function by binding to MHC proteins on antigen-presenting cells (APCs) and to the TCR on adjacent T helper cells, connecting signaling molecules and thus leading to T cell activation. SAg does not react with the unique antigen-binding site on the TCR, formed by the alpha and beta chains, but only with the outer surface of the V segment of the beta chain. All T lymphocyte clones with a specific beta chain structure in their TCRs (regardless of the alpha chain that makes up the TCR molecule) are activated. This simultaneous interaction results in massive activation of the immune system, accompanied by intense proliferation of CD4+ or CD8+ T lymphocytes. Compared to the response to a normal antigen, where approximately 10^−3^–10^−4^% of T lymphocytes are activated, SAg can activate up to 20% of the total human T lymphocyte pool. A large number of activated T lymphocytes secrete large quantities of cytokines, including interferon gamma (IFN-gamma), which activates macrophages. These macrophages then overproduce proinflammatory cytokines, such as IL-1, IL-6, and TNF-alpha (normally released locally in small amounts). However, when TNF-alpha is present in the blood and in large amounts, it can cause serious and life-threatening symptoms, including septic shock and multiple organ failure [43,45,46,47]. Human specificity TCR Vbeta for staphylococcal superantigens is presented in Table 3 [46,47,48,49,50,51,52,53,54,55,56,57,58,59,60,61,62,63,64,65].

SAgs include the toxin TSST-1 and enterotoxins and enterotoxin like proteins SEA, SEB, SEC, SEC-1, SED, SEE, SEG, SEH, SEI SElJ, SEK, SEL, SEM, SEN, SEO, SEP, SEQ, SER, SES, SET, SElU, SElV, SElW, SElX, SEY, SElZ, SE01, SE02, SEl26, and SEl27 [66].

*S. aureus* and *S. pyogenes* superantigens were classified into four of the five phylogenetic groups [44,51,52].

Group I: TSST-1, TSST-ovine, SET, SElX, SEY.

Group II: SEB, SEC, SEC1, SEC2, SEC3, SEG, SER, SElU, SElZ and *S.pyogenes*: SpeA, SSA.

Group III: SEA, SED, SEE, SEH, SElJ, SEN, SEO, SEP, SES, SElW and *S.pyogenes* SpeH, SePE-H.

Group IV superantigens: *S. pyogenes* SpeC, SpeG, Spe-Gdys, SpeJ, SpeK, SpeL, SpeM, SePE-M, SePE-L, SMEZ-1, SMEZ-2, SDM.

Group V: SEI, SEK, SEL, SEM, SEQ, SElV and *S.pyogenes* SpeI, SePE-I.

TSST-1, belonging to group I SAg, binds to MHC class II via a low-affinity N-terminal binding domain that is peptide-dependent, and binds to TCR Vbeta via two independent regions in both CDR2 and the framework region (FR) 3 [44,67].

Group II SAgs also bind to the alpha chain of MHC class II via an N-terminal low-affinity binding domain, but unlike group I, this binding is peptide-independent [68].

Group III SAgs are able to form cross-links with MHC class II molecules via a low-affinity site, like group II, as well as via a zinc-dependent high-affinity site binding to MHC class II, located in the beta-grasp domain of the SAg [64].

SAgs from group IV occur exclusively in *Streptococcus* and contain a high-affinity MHC class II binding domain similar to group III. SpeC binds to Vbeta. They make multiple contacts with both the side chain and main chain atoms of Vbeta2.1, and all three CDR loops are involved, as well as hypervariable (HV) region 4 and FR3 [44,69].

SAgs from group V have loop extensions between the third alpha helix and the eighth beta sheet (which we call the alpha3–beta8 loop). This homologous extension of approximately 15 amino acids is not found in other SAg groups [44].

Staphylococcal superantigens can be divided into two groups based on their specific binding to alpha chains (low affinity) and beta chains (high affinity dependent on Zn^2+^) of MHC class II cells. Group III, IV, and V superantigens bind to alpha and beta chains of MHC class II: SEA, SED, SEE, SEH, SEI, SElJ, SEK, SEL, SEM, SEN, SEO, SEP, SEQ, SES, and SElV. Group III enterotoxins SEA, SED, SEE, SEH, SElJ, SEN, and SEP contain a cystine loop with nine amino acids. Only staphylococcal superantigens of groups I and II bind to the alpha chain of MHC class II: TSST-1, SElX, TSST-bovine SEB, SEC, SEG, SER, SET, and SElU. Group II enterotoxins SEB, SEC, SEG, SElU, and SElW (SElU2) have a cystine loop with a variable sequence of 10–19 amino acids separating the cysteine residues [51,52,66].

### 3.5. Biofilms

Biofilms limit the access of macrophages, lymphocytes, antibodies, and antibiotics to *S. aureus* cells that are part of biofilms. Cells present in biofilms may show tolerance to vancomycin. Tolerance is defined as the lack of susceptibility of bacteria to the bactericidal action of an antibiotic, and the minimum bactericidal concentration is more than 32 times higher than the minimum inhibitory concentration (MIC > 32MIC). Cells in the deeper layers of the biofilm, which undergo anaerobic metabolism, are resistant to antibiotics from the aminoglycoside group [70,71,72]. A more detailed discussion of biofilms is presented later in this paper.

### 3.6. Capsular Polysaccharide (CP)

In *S. aureus*, an important opportunistic pathogen, the expression of the polysaccharide capsule significantly contributes to its ability to cause invasive diseases [73,74,75], and the capsules themselves are considered important factors of pathogenicity [76]. Capsules in *S. aureus* are not as important as those in *Streptococcus pneumoniae*, for example, where non-capsulated strains become non-pathogenic. In *S. aureus*, however, one of the most pathogenic clones, the USA300 clone, is non-capsulated. *S. aureus* capsules can protect bacterial cells from phagocytosis and help bacteria evade the host’s immune system. CP1 and CP2 capsules have the strongest protective effect but are relatively rare. Most *S. aureus* strains have CP5 and CP8 capsules, which exhibit moderate anti-phagocytic activity. The capsules are synthesized during the logarithmic growth phase of the bacteria, i.e., during the first phase of infection. The regulation of expression is described in Chapter 9 [77,78,79].

To date, 11 serotypes of *S. aureus* capsules have been isolated, from CP1 to CP11 [80], and the chemical structures of four of them have been determined: CP1, CP2, CP5, and CP8. Their chemical formulas are shown below:

CP1: ⟶4)-alpha-D-GalNAcAp-(1⟶4)-alpha-D-GalNAcAp-(1⟶3)-alpha-D-FucNAcp-(1⟶
CP1 also contains taurine, which is linked to every fourth D-GalNAcA unit by an amide bond.
CP2: ⟶4)-beta-D-GlcNAcAp-(1⟶4)-beta-D-GlcNAcAp-(L-alanyl)-(1⟶
CP5: ⟶4)-beta-D-ManNAcAp-(1⟶4)-alpha-L-FucNAcp-(3OAc)-(1⟶3)-beta-D-FucNAcp-(1⟶
CP8: ⟶3)-beta-D-ManNAcAp-(4OAc)-(1⟶3)-alpha-L-FucNAcp)-(1⟶3)-alpha-D-FucNAcp)-(1⟶
Abbreviations: GalNAcAp, N-acetyl-galactosaminuronic acid; FucNAcp, N-acetyl-D-fucosamine; GlcNAcAp, N-acetyl-glucosaminuronic acid; ManNAc, *N*-acetyl mannosaminuronic acid; OAc, O-acetyl.

The synthesis of *S. aureus* capsules is controlled by the *cap* operon, which contains 16 genes (from *capA* to *capP*) encoding various enzymes involved in CP biosynthesis and transport [81]. The expression of the cap gene cluster is tightly controlled during *S. aureus* growth, undergoing repression before the exponential phase, showing primary expression in the late exponential phase, and showing maximum production in the stationary phase [82]. The main promoter of the PsigB capsule operon is controlled by the Sigma B (SigB) regulator, and the second weak promoter PsigA is regulated by Sigma A (SigA) [83]. Positive regulation of the cap operon is exerted by the MgrA, SpoVG, and RbsR regulatory systems, while negative regulation is exerted by Rot, CodY, and SaeSR. MgrA binds directly to PsigA, and other regulators such as SigB, SbsDC, TCS, AgrCA, and ArlSR can influence CP biosynthesis indirectly through the regulation of MgrA. Over 90% of clinically isolated *S. aureus* strains produce type 5 (CP5) or type 8 capsules (CP8) [84,85,86]. *S. aureus* USA300 does not have a capsule due to several conservative mutations in the *cap5* locus [87]. Among the MRSA clones dominant worldwide, CP8 capsules have ST1, ST30, ST59, ST80, and ST239; CP5 capsules have ST5 and ST22 [81].

## 4. Surface Proteins

Surface proteins include the proteins discussed earlier in this paper that protect bacteria from the immune system, such as Spa, Sbi, Scn (SCIN), eqSCIN, Ecb, Chp, Vwb, and proteins responsible for colonization and biofilm formation, such as FnbpA, FnbpB (fibronectin binding protein A and B), ClfA, ClfB (clumping factor A and B), SdrC, SdrD, SdrE (serine-aspartate repeat-containing protein C, D or E), IsdA (iron-regulated surface determinant protein A), Atl (major autolysin and adhesin), Aaa (two-domain protein autolysin/adhesin), Eap (extracellular adherence protein), Emp (extracellular matrix protein-binding adhesin), Ebh (extracellular matrix-binding protein), EbpS (elastin binding protein S), Cna (collagen binding adhesin), Bap (biofilm-associated protein), Sas G, and SasX (*S. aureus* surface protein G or X). *S. aureus* surface proteins that may play a role in pathogenicity mostly belong to the MSCRAMM (microbial surface components recognizing adhesive matrix molecules) group.

Many surface proteins such as Spa, FnbpA, FnbpB, ClfA, ClfB, SdrC, SdrD, SdrE, Eap, Fib, and SasG are anchored in the cell wall by covalent binding to peptidoglycans. The protein SrtA (membrane sortase A) participates in this process. The polymorphism of the *spa* gene is used for epidemiological typing of *S. aureus* and determining the spa type, while the polymorphism of the *sspA*, *spa*, *sdrC*, *sdrD*, *sdrE*, *clfA*, and *clfB* genes is used for strain typing in the MLVA system (*S. aureus* multiple-locus variable-number tandem-repeat analysis) [11,36,88,89,90,91,92].

The most important surface proteins responsible for colonization and biofilm formation are presented in Table 4 according to previous studies [16,17,19,37,93,94,95,96].

## 5. *S. aureus* Biofilms

A biofilm is a complex multicellular structure of bacteria surrounded by a layer of organic and inorganic substances produced by these microorganisms. It adheres to both biological and abiotic surfaces. Biofilm protects the bacteria that form it from environmental factors, the immune system, and antibiotics. Biofilms contribute to the pathogenesis of chronic diseases, especially infections associated with the use of catheters, drains, implants, and surfaces in hospital wards. Biofilm can be formed by a single species of bacteria, several species of bacteria, or bacteria and fungi (mixed biofilms). The process of biofilm formation by *S. aureus* can be divided into several stages. *S. aureus* cells begin to synthesize surface proteins, which is preceded by the blocking of the Agr regulatory system. Initial adhesion and attachment begin, and individual cells or aggregates adhere to the surface. Aggregation occurs, with cell division and proliferation, as well as the production of EPS (extracellular polymeric substances), structuring and maturation of the biofilm, in which *S. aureus* cells coexist in polymicrobial interactions. The final stage involves biofilm dispersion, with cells detaching from the aggregated biofilm to a planktonic state, allowing the biofilm to spread within the body. This stage is preceded by the activation of the Agr system. Agr activates the synthesis of enzymes, mainly proteases, hydrolases, and autolysins. Some cells are lysed, leading to the formation of eDNA, which is used to create new biofilms [88,97,98]. *S. aureus* is a relatively anaerobic bacterium, which means that it can alternatively carry out aerobic and anaerobic metabolism. *S. aureus* cells growing in biofilm are divided into four zones representing different metabolic states: aerobically growing bacteria (located in the oxygenated and nutrient-rich outer layer), anaerobically growing bacteria undergoing fermentation (located in the nutrient-poor inner layer), dormant anaerobic bacteria (located in the anaerobic layer with slow growth and inactive metabolism), and dead bacteria. Biofilm protects bacteria and alters the distribution of antibiotics within the biofilm, often preventing their access. This is especially true for large-molecule antibiotics. In addition, inside the biofilm, some bacteria are metabolically inactive, while others undergo strictly anaerobic metabolism, which prevents certain drugs from working. Furthermore, negatively charged polysaccharides can block the penetration of positively charged antibiotics. All of this affects changes in MIC and MBC values [99,100,101]. Current anti-biofilm strategies include: a combination of physical destruction of biofilm (surgical or ultrasonic) + antibiotic treatment; agents targeting the inhibition or destruction of EPS (extracellular polymeric substances) such as cyclic di-GMP (cdGMP), cyclic di-AMP (cdAMP), enzymes that break down EPS (glucan hydrolases, dispersin B, serine protease, DNA-za); blocking quorum sensing detection signaling via quorum quenching factors; recombinant bacteriophages; use of nanoparticles (TiO2 or silver); use of the bioelectric effect.

### 5.1. PIA (Polysaccharide Intercellular Antigen)-Dependent Biofilm

**Figure 1:**
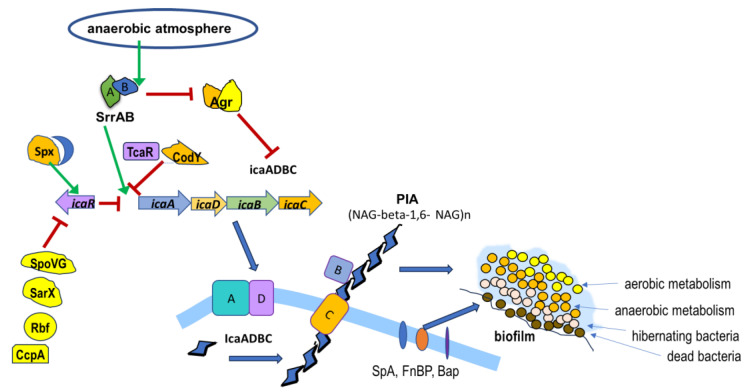
The mechanisms involved in PIA-dependent biofilm formation. Abbreviations: PIA, polysaccharide intercellular antigen; Bap, biofilm-associated protein; FnBP, fibronectin binding protein; Spa (SpA), *Staphylococcus aureus* immunoglobulin G binding protein A; SrrAB, SpoVG, SarX, Rbf, CcpA, regulatory systems activating IcaADBC; Agr, CodY, Spx, regulatory systems inhibiting IcaADBC.

PIA-dependent biofilm is most produced by HA-MRSA strains in which the Agr regulatory system is blocked. PIA (PNAG, poly-N-acetylglucosamine) is a glycan with repeating 2-acetamido-2-deoxy-D-glucopyranosyl residues linked by beta-1,6 bonds. PIA formation enables intercellular adhesion, aggregation, and attachment to abiotic surfaces for *S. aureus*. PIA synthesis depends on the *ica* (intercellular adhesin) operon. The expression of the *icaRADBC* operon depends on several regulatory systems: SrrAB (respiration response regulator), which inhibits the Agr system, enabling the expression of surface proteins (Spa, FnBP, Bap), and the Rbf (protein regulator of biofilm formation), which inhibits the expression of *icaR* (repressor of the *icaADBC* operon) via the SarX regulator [70]. Inhibition of biofilm formation is caused by the active Agr system and may also result from inhibition of Rbf expression by the LuxS/AI2 (quorum sensing/autoinducer 2) regulatory system, activation of *icaR* by SpoVG (global stress response regulator), and by TcaR and CodY, which inhibit the expression of *icaADBC* [102,103]. The *ica* operon is also regulated by SarA and SigB [104]. The expression of the *icaADBC* operon results in: IcaA (transmembrane N-acetylglucosaminyltransferase), which together with IcaD forms N-acetylglucosamine oligomers. IcaC then transfers these polysaccharides from the cell surface, and IcaB deacetylates the poly-N-acetylglucosamine molecules, contributing to the adhesion of these polymers to the outer cell surface and the formation of biofilm [70,104,105,106]. The Bap protein works with PIA to promote aggregation between cells during biofilm formation, while the Aap protein is associated with biofilm maturation accumulation processes [93,107]. The mechanisms involved in PIA-dependent biofilm formation are shown in Figure 1.

### 5.2. PIA-Independent Biofilm

PIA-independent biofilm is most commonly produced by HA-MRSA strains in which the Agr regulatory system is blocked and the *icaADBC* operon is inactive. The Rsp regulator can also inhibit biofilm formation by inhibiting the expression of certain surface proteins such as FnbpA, FnbpB, ClfA, Spa, and SasG [108]. The aggregation of *S. aureus* planktonic cells occurs via expressed surface proteins, which are also responsible for the adhesion of the forming biofilm. According to previous studies [109,110,111,112], these include binding proteins:

- fibrinogen: FnbpA, FnbpB, ClfA, ClfB, SdrE, IsdA forming covalent bonds and Atl, Aaa, Eap, Emp forming non-covalent bonds
- fibronectin: FnbpA, FnbpB, IsdA forming covalent bonds and Atl, Aaa, Eap, Emp, Ebh forming non-covalent bonds
- elastin: FnbpA, FnbpB (covalent bond) and EbpS (non-covalent bonds)
- collagen: Cna forming covalent bonds and Eap, Emp forming non-covalent bonds;
- covalently bone sialoprotein: Bbp, SdrC, SdrD
- covalently glycolipids: Pls
- non-covalently vitronectin: Atl, Aaa, Eap, Emp
- endoprosthesis polystyrene: Bap, SasC (covalent bonds) and Atl (non-covalent bond)

Activation of the interaction between the Spa protein and vWF glycoprotein may promote bacterial adhesion to damaged blood vessels [113].

### 5.3. Biofilm Formed from Extracellular DNA (eDNA)

Activation of *cidABC* operon expression (autolysins) and proteases stabilized by *sarA* and reduced expression of the *igr* gene (murein hydrolase regulator) cause autolytic activity of a subpopulation of cells undergoing anaerobic metabolism, which results in the release of genomic DNA, and, with reduced expression of the *nuc* gene, contributes to cell adhesion during biofilm maturation. The chemical nature of the long, electrically charged DNA molecule modulates the properties of the cell surface and promotes adhesion between cells and the surface, as well as playing a structural role in the *S. aureus* biofilm matrix [114].

## 6. Toxins

Toxins are a key virulence factors, causing many clinical symptoms in infected individuals. Toxins protect bacteria from the immune system and help spread infection. *S. aureus* produces many toxins with different mechanisms of action, including (1) Pore-forming toxins (Hla, Hlb, leukotoxins, PSM), (2) Toxic shock toxin (TSST1), (3) Epidermolytic toxins, and (4) Enterotoxins.

### 6.1. Pore-Forming Toxins (PFTs)

#### 6.1.1. Hemolysin alpha (Hla, alpha-Toxin)

Hla, a protein (33 kDa; premature form 319 aa; mature form 293 aa), encoded by the chromosomal *hla* gene, is present in 95% of clinical strains of *S. aureus*. Hla synthesis is stimulated by *agr*RNAIII and inhibited by SarA, SarR, SarT, SarH1, and Rot. In an infected organism, Hla binds to the transmembrane protein ADAM10 (a desintegrin and metalloproteinase 10), which is its receptor. It then oligomerizes in the plasma membrane to form heptamers, resulting in the formation of preliminary pores and then transmembrane channels [66,115]. ADAM10 is found in erythrocytes, platelets, epithelial and endothelial cells, keratinocytes, pneumocytes and human B and T lymphocytes [116,117,118,119,120,121,122,123]. After binding to Hla, ADAM 10 is activated and degrades E-cadherin, causing the loss of homotypic interactions between cadherin molecules of adjacent cells in adhesive junctions. This leads to impaired epithelial structure and promotes the spread of *S. aureus* in the infected organism [124]. Pores on the surface of the host cell allow the loss of intracellular K^+^ and the influx of extracellular Ca^2+^, leading to cell death [125]. Released circulating Hla can activate the sphingomyelinase/ceramide pathway [126], leading to degradation of tight junctions of endothelial cells in the lungs, causing severe pulmonary edema [127]. Patients may develop pulmonary edema even with proper antibiotic treatment after the bacteria have been killed. Furthermore, activation of the sphingomyelinase/ceramide pathway is also associated with inflammasome activation, resulting in the release of IL-1beta and TNF-alpha [128]. Research into non-antibiotic treatment methods is ongoing. Trials conducted on animal models using Hla-neutralizing antibodies or ADAM10 inhibitors reduced early VE-cadherin cleavage, skin hypoxia, and skin necrosis. In intensive care units patients undergoing mechanical ventilation who have *S. aureus* colonization of the lower respiratory tract, the incidence of *S. aureus* pneumonia within 30 days was not significantly lower after treatment with anti- alpha toxin monoclonal antibody -suvratoxumab than after placebo. Despite these negative results, monoclonal antibodies remain a promising therapeutic option for reducing antibiotic use, which requires further research and analysis.

#### 6.1.2. Hemolysin beta (Hlb, beta-Toxin, Sphingomyelinase C)

The Hlb protein encoded by the chromosomal *hlb* gene is produced by 80–90% of clinical strains of *S. aureus* [129]. In addition, the *hlb* gene contains a site of integration for many bacteriophages (in the chromosome of the MRSA252 strain, the *hlb* coding sequence is interrupted by the integration of the phiSa3 (st.252) prophage after residue 61 [37]. Hlb causes lysis of human erythrocytes, which are moderately sensitive, as well as keratinocytes, monocytes, and T lymphocytes. Hlb stimulates the production of proinflammatory cytokines in human monocytes, but inhibits IL-8 production and cell adhesion molecule expression in human endothelial cells; thus, the toxin prevents leukocyte migration through endothelial cells [66,130,131].

#### 6.1.3. Leukotoxins

Leukotoxins are classified as synergohymenotropic toxins due to the interaction of two proteins, one from the F (Fast) group and one from the S (Slow) group. Theoretically, any F protein can form a leukotoxin with any S protein, but they are most often encoded in pairs: LukF-PV/LukS-PV, HlgA/HlgB, HlgC/HlgB (gamma hemolysin, LukF/LukS, LukF-R/LukS-R), LukD/LukE, LukG/LukH (LukA/LukB), isolated from human infections, and LukQ/LukP and LukF’ (LukF-PV)/LukM, isolated from *S. aureus* in animal infections [42,132,133,134].

The genes encoding leukotoxins are found in the chromosome and may be present in the prophage genome and in pathogenicity islands (SaPI). The production of LukF-PV/LukS-PV may be the result of lysogenic conversion by the phages phiPVL, phi108PVL, phiSLT, phiSa2MW, phi Sa2USA, and phiSa2958 [135], LukQ/LukP is encoded by the prophage phiSaeg1, and the LukD/LukE encoding genes are found in SaPIn3 [42,136].

The formation of pores in the cytoplasmic membrane of target cells by leukotoxins consists of several stages. The S subunit of leukotoxin in monomer form recognizes and binds to a surface receptor on target cells. Human CXCR1, CXCR2, CCR2, and CXCR4, as well as the Duffy antigen receptor for chemokines (DARC), are receptors for HlgA [137,138]. This is followed by dimerization with the F subunit and oligomerization of SF and formation of a primary pore (prepore), followed by insertion of a beta-barrel transmembrane channel into the cell membrane. A beta-barrel pore is formed, which destroys the target cell membrane [139,140,141,142]. Leukotoxin LukAB (LukGH), which is secreted in the form of dimers, deviates from this mechanism of action. LukAB (LukGH) binds to integrin CD11b, which is a receptor on the surface of the target cell. After binding to the receptor, LukAB (LukGH) dimerizes with additional LukAB (LukGH) leukocidin dimers to form an octameric prepore. The prestem domains of the prepore extend to form a beta-barrel pore, which ultimately destroys the target cell membrane.

#### 6.1.4. Phenol Soluble Modulins (PSM, Cytolytic Toxins)

The name of this group of toxins comes from the fact that three peptides, PSMalpha, PSMbeta, and PSMγ (Hld), were isolated by hot phenol extraction from culture filtrates [143,144]. PSM toxins include PSM alpha 1, PSM alpha 2, PSM alpha 3, PSM alpha 4 encoded by the chromosomal *psm alpha* operon, PSM beta 1 and PSM beta 2 toxins encoded by the chromosomal *psm beta* operon, and Hld (hemolysin delta) toxin encoded in the region transcribed from the P3 promoter and forming the *agr*RNAIII transcript [38,66,145]. PSMs are short peptides with a size of 20–22 aa (PSM alpha), 44 aa (PSM beta), 45 aa (Hld). PSM alpha are positively charged, PSM beta are negatively charged, and delta toxin is neutral. PSM expression is activated by the phosphorylated AgrA protein of the Agr regulatory system and can be inhibited in HA-MRSA strains by the PSM-mec factor found in SCCmec type II, III, and VIII cassettes [146]. PSMs play an important role in cell lysis (including intracellular lysis of neutrophils and osteoblasts), biofilm formation, and immune modulation. PSMs attach to the cytoplasmic membrane in a non-specific manner. This attachment can lead to membrane disintegration. PSMs aggregate into oligomers and form transient pores [132,133,134].

PSM in nanomolar concentrations, acting mainly through the FPR2 receptor present on macrophages, neutrophils, monocytes, and immature dendritic cells, initiate proinflammatory responses, including neutrophil chemotaxis, leukocyte activation, and cytokine production including IL-8 [147,148,149]. PSMalpha induces keratinocyte death and stimulates the release and secretion of IL-1alpha and IL-36alpha alarmins. Myd88 (interleukin receptor signal adaptor) participates in this process (IL-1Ri IL-36R), leading to IL-17-mediated skin inflammation [150]. Delta toxin has also been shown to be a potent inducer of mast cell (MC) degranulation in atopic dermatitis (AD) [151].

### 6.2. Toxic Shock Toxin (TSST-1)

TSST-1 was first described as enterotoxin F, and later renamed TSST-1 [152]. TSST-1 is a superantigen and has no emetic activity. T/APC lymphocytes activated by the toxin lead to the release of various cytokines and cause immunosuppression of T and B lymphocytes, fever, decreased blood pressure, rash, vascular disorders, toxic shock syndrome (menstrual and non-menstrual), and multiple organ failure. TSST-1 increases epithelial permeability, facilitating the penetration of toxins and other pathogens into tissues and the bloodstream. The epithelial dysfunction caused by TSST-1 increases the susceptibility of the skin to secondary infections, including those caused by the HSV virus, thereby exacerbating the inflammatory response [66,153,154,155]. TSST-1 is encoded by the *tst* gene, initially located in transposon-like elements, and later found to be present in pathogenicity islands: SaPI2, SaPIn1, SaPIj, SaPISaitama2. TSST-1 expression is activated by the Agr and SaeRS regulatory systems and inhibited by the SarA, Rot, and SigB regulators [10,136,155,156,157,158,159].

### 6.3. Epidermolytic Toxins (Exfoliatins, ET)

Five epidermolytic toxins have been described: ETA, ETB, ETC, ETD, and ETE, but only ETA and ETB were produced by *S. aureus* isolated from humans, while the others originated from animal biotypes of *S. aureus* [66]. The *eta* gene has been described in the genome of the prophage phiETA (43,081 bp) in strain E1 [160,161] and in SaPI2R st. OC03 [162], and the *etb* gene was described in a plasmid isolated from the TYE strain [161] and in the chromosome of the JP080 strain [16]. ETs occur in approximately 5% of *S. aureus* strains and are most commonly isolated in sequence types ST121, ST15, ST109, and ST582 [163,164]. ETs are glutamate-specific serine proteases of the chymotrypsin family. ETs recognize and induce hydrolysis of desmosome cadherins in the superficial layers of the skin. By interacting with human desmoglein 1 (Dsg1), ETs can cleave keratinocyte junctions and cell–cell adhesion in the host epidermis, inducing blister formation. ET synthesis by *S. aureus* strains infecting the skin can cause local symptoms such as bullous impetigo. In newborns and infants, and rarely in adult patients with compromised immunity, the toxin can act systemically, causing staphylococcal scalded skin syndrome (SSSS) [165,166,167,168].

Table 5 presents toxins from the group of hemolysins, PSM, leukotoxins, exfoliatins, and exotoxins according to previous studies [16,37,38,42,135,136,145,161,162,169,170,171,172].

### 6.4. Enterotoxins

According to the nomenclature proposed by the INCSS (International Nomenclature Committee for Staphylococcal Superantigens) [173], staphylococcal enterotoxin (SE) must exhibit emetic activity after oral administration in primates. In cases where the toxin does not exhibit emetic activity in primates or where this has not been tested, the toxin is designated as SEl (staphylococcal enterotoxin-like). SE and SEl cause food poisoning and are classified as superantigens. The SE and SEl encoding genes are found in the *S. aureus* chromosome (they can form an enterotoxin gene cluster, egc), plasmids, pathogenicity islands, and the prophage genome. Table 6 presents selected characteristics of SE and SEl according to previous studies [10,23,39,66,136,158,162,174,175,176,177,178,179,180,181,182,183,184,185,186,187,188,189,190,191,192,193,194,195,196,197].

## 7. Enzymes and Other Proteins

*S. aureus* also produces many enzymes and other proteins that help it to survive and spread in the host organism classified as virulence factors, such as Coa (coagulase, staphylocoagulase, coagulation mediator), Nuc (thermonuclease), SplA-SplF and V8 (serine proteases and serine like proteases), ScpA (cysteine protease), Aur (zinc metalloproteinase aureolysin), Geh1 and Geh2 (Lip1 and Lip2; lipase1 and lipase2), HY-ase (hyaliauronate lyase), ceramidase [198], SrtA (sortase) and other protein factors vWbp (von Willebrand binding protein) [199], EdinA-EdinC (epidermal cell differentiation inhibitor), and EDIN-like exotoxin [200].

### 7.1. Coagulase (Coa, Staphylocoagulase)

Coagulase is an extracellular protein that has the ability to bind to both prothrombin and fibrinogen. The coagulase-prothrombin complex (staphylothrombin) can cause fibrinogen coagulation without proteolytic cleavage of prothrombin, in a manner different from natural coagulation [201]. Coagulase also acts as an extracellular adhesive factor and is a virulence factor in lung infections [202]. The polymorphic chromosomal *coa* gene encodes a coagulase protein with a variable number of amino acids in different S. aureus strains, e.g., GenBank acc X17679 (636 aa), D00184 (715 aa), X16457 (632 aa), and AP017922 (504 aa). The coagulase protein has a variable number of tandem repeats in its terminal region. The *coa* gene is negatively regulated by *agr*RNAIII [157,201,203,204].

### 7.2. Thermonuclease

Thermonuclease (Nuc, EC3.1.31.1.) is an extracellular protein encoded by the chromosomal genes nuc: *nuc1* and *nuc2*. Usually, two forms of the enzyme are present in a single *S. aureus* strain, a larger and a smaller one, encoded in different locations on the chromosome, e.g., GenBank AP017922 st. st.JP080, 228 aa and 177 aa. Nuc1 is involved in evading immune responses, and both proteins participate in biofilm formation [205].

### 7.3. Proteases

*S. aureus* proteases participate in various stages of invasive diseases. They break down host proteins, e.g., ScpA breaks down fibrinogen and collagen, Atl degrades peptidoglycan hydrolase, and Aur and ScpA selectively degrade bacterial virulence factors, thus influencing the course of infection. *S. aureus* secretes extracellular serine proteases and serine protease-like proteins (e.g., SspA, HtrA, SplA, SplB, SplC, SplD, SplE, and SplF) and cysteine proteases such as staphopain A (ScpA) and SspB; the metalloprotease/aureolysin Aur. *S. aureus* also produces intracellular proteases that influence the quality control of cellular proteins, stress responses, virulence regulation, and antibiotic resistance, e.g., caseinolytic protease P (ClpP) and carboxyl protease (CtpA). Among extracellular proteases, only Spl enzymes encoded by splA-F genes do not require proteolytic activation. In contrast, Aur, ScpA, SspA, and SspB proteases are produced as zymogens or proenzymes that require proteolytic activation [115,206,207].

### 7.4. EDIN (Epidermal Cell Differentiation Inhibitor)

EDIN is an inhibitor of epidermal cell differentiation, inhibiting the final differentiation of cultured keratinocytes. Its mechanism of action depends on the activity of ADP-ribosyltransferase, which modifies GTP-binding Rho proteins (RhoA, RhoB, and RhoC). ADP-ribosylation of Rho proteins causes the breakdown of the actin cytoskeleton in intact cells. The EDIN protein gene (247 aa) is located on chromosome (GenBank M63917) [208].

## 8. Iron Uptake Systems

The predominant form of iron in humans is heme, which consists of a tetrapyrrole ring. Heme is bound by heme proteins, the most common of which is hemoglobin. This is sequestered intracellularly in erythrocyte. In addition, iron is found in the cytochromes of all cells and in myoglobin in myocytes. Iron is also found in extracellular proteins such as haptoglobin (a tetrachin alpha2 beta2 glycoprotein), which binds free hemoglobin after hemolysis, in the iron exporter ferroportin, transferrin, lactoferrin, hemopexin, and others. Over 90% of iron in the human body is found intracellularly, which forces extracellular pathogens to use mechanisms that release iron sources [209]. Iron uptake by *S. aureus* is regulated by the Fur regulator (iron uptake regulatory protein; GenBank AF118839; 149 aa). In the presence of iron, Fur binds to a DNA sequence known as the fur box, inhibiting the transcription of many genes. Under conditions of iron starvation, Fur repression is relaxed, resulting in the upregulation of many genes involved in iron acquisition, glycolysis, and virulence. The lack of Fur repression directly or indirectly activates the synthesis of virulence factors: hemolysins, hydrolases, cytolysins, nucleases, proteases, and lipases, chemotaxis inhibitory protein (CHIP), staphylococcal complement inhibitor (SCIN), coagulase, exotoxins Ssl 1, Ssl 2, Ssl 6, Ssl 7, Ssl 9, and Ssl 11, exotoxins, biofilm-forming proteins Eap and Emp, and operons responsible for iron uptake [209,210,211].

### 8.1. Systems That Extract Iron from Hemoglobin and Heme

The Isd system comprises proteins encoded by the *isdBACDEF* operon (e.g., JP080; GenBank acc. BX571856) [37], the heme oxygenase proteins IsdG (staphylobilin-forming heme oxygenase IsdG) and IsdI, and sortases A and B. The isdBACDEF operon encodes the heme-binding proteins IsdA and IsdC, the hemoglobin receptor IsdB, the hemoglobin-haptoglobin receptor IsdH, and the membrane transporter IsdEF. IsdA, IsdB, and IsdH are anchored in the cell wall by sortase A, and IsdC by sortase B [212,213]. The Isd system consists of two cytoplasmic heme oxygenases, IsdG and IsdI [212]. Isd proteins enable *S. aureus* to use hemoglobin as a source of iron. IsdA, IsdB, IsdH, and IsdC contain NEAr (NEAT) iron transporter domains. NEAT domains are conserved amino acid sequences that mediate the binding of heme and heme proteins [214]. Heme iron is bound in the clefts by a single axial tyrosine ligand [215]. IsdB and IsdH use NEAT domains to initiate the process by binding hemoglobin or hemoglobin-haptoglobin, respectively. IsdB has two NEAT domains, and it is the second domain that transfers heme to a single NEAT domain of IsdA or IsdC. Heme is directly transferred from IsdA to IsdC in an affinity-dependent manner [216]. IsdA is unable to transfer heme to IsdE, but heme can pass directly from IsdC to IsdE [217]. Heme then crosses the cell membrane via the ABC transporter IsdEF. Upon entering the cytoplasm, heme is degraded by the heme oxygenases IsdG and IsdI [218,219,220]. *S. aureus* is primarily a human pathogen, and the *S. aureus* hemoglobin receptor IsdB binds human hemoglobin more effectively than hemoglobin from other animal species [221].

Outside the Isd system, heme can be transported into the cytoplasm of *S. aureus* cells by the HtsBC transporter [218].

### 8.2. Systems That Extract Iron from Transferrin and Lactoferrin

Siderophores target and remove iron present in transferrin and lactoferrin [209,222,223]. The production of siderophores is stimulated by the blocking of the Fur regulator in the event of iron deficiency [224,225]. *S. aureus* synthesizes two siderophores, staphyloferrin A and staphyloferrin B. They are synthesized by the NIS (nonribosomal peptide synthesis independent siderophore) pathway and then secreted into the extracellular environment. The siderophore-iron complexes are then transported back into the cytoplasm by ABC HtsABC transporters [226]. Staphyloferrin A is synthesized by the *sfaABC* and *sfaD* operons separated by the Fur box region (Fur repressor binding site) [225,227,228]. The genes encoding staphyloferrin B synthesis are located in the *sbnABCDEFGHI* operon, which is regulated by the Fur regulator [226]. After binding iron, both staphyloferrin A and staphyloferrin B, as well as hydroxamic acid-type siderophores, are transported to the cytoplasm by the ABC transporters HtsABC, SirABC, FhuBGC2-D1/D2, and SstABCD (staphylococcal siderophore transporter) [223,226]. *S. aureus* does not produce hydroxamic siderophores, but can utilize certain exogenous Fe (III)-hydroxamic acids, such as ferrochrome, desferrioxamine B, coprogen aerobactin, and rhodoturolic acid. The uptake of hydroxamic siderophores is mediated by the Fhu transporter, encoded by the *fhuCBG* operon, regulated by Fur [223].

## 9. Regulatory Systems

The expression, or lack of expression, of many virulence genes is determined by regulatory systems. These include TCS (two-component systems) and transcription factor-based systems. TCS include Agr, ArlRS, SaeRS, SrrAB, and LuxS/AI-2 QS. Regulatory transcription factors systems include Fur, Rbf, Rsp, SRR42, Rot, MgrA, SigB, Rpx, SarA, SarR, SarS, SsarT, SarU, SarV, SarX, and SarZ.

### 9.1. Two-Component Systems (TCS)

Agr (global Agr regulatory system)

**Figure 2:**
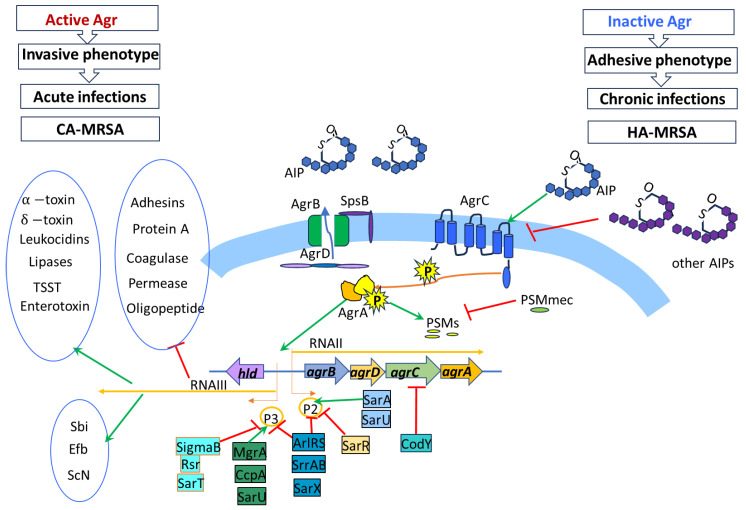
The most important impact pathways related to the Agr regulatory system. Abbreviations: RNAII, transcript of the Agr region from promoter P2, containing the genes *agrBDCA*; RNAIII, transcript of the Agr region from promoter P3, contains the *hla* gene; PSM, phenol soluble modulin; PSMmec, repressor of PMS and Agr; Sbi, *S. aureus* binder of IgG; Efb, extracellular fibrinogen binding protein; Scn, staphylococcal complement inhibitor; MgrA, CcpA, SarU, SarA, regulatory systems activating Agr; Sigma B, Rsr, SarT, SarR, regulatory systems inhibiting Agr.

The Agr system (*agrACDB*/RNAIII, accessory gene regulator, quorum sensing system) is the most important of the many systems that regulate gene expression in S. aureus. Agr activation is associated with the production of toxins and other factors that cause acute, spreading infection. Conversely, lack of Agr activation is associated with biofilm production and the development of chronic infection (Figure 2).

The Agr system covers a region of approximately 3 kb in which the following genes are located: 

*agrA* (AgrA, accessory gene regulator protein A, 238 aa GenBank acc. AP017922, st. JP080);

*agrC* (AgrC, histidine kinase, 430 aa, GenBank acc. AP017922, st. JP080);

*agrD* (*crtN*) (AgrD, staphylococcal accessory gene regulator protein D, 46 aa, GenBank acc. AP017922, st. P080);

*agrB* (AgrB, accessory gene regulator protein B, 207 aa GenBank acc. AP017922, stJP080; membrane protein putatively involved in post-translational modification of the autoinducing quorum-sensing peptide).

*hld* (Hld, delta-hemolysin, 45aa, GenBank acc. AP017922, st. JP080) and promoters P2 and P3. The *agrD* gene encodes a peptide that is post-translationally converted into AIP (autoinducing peptide, containing a thiolactone ring, 4 types: AIP1, 2, 3, or 4). *S. aureus* strains with a specific AIP type (one strain synthesizes only one AIP type) are classified as agrI, agrII, agrIII, or agrIV. The amino acid sequence of AIP molecules is presented below [229,230].

AIP1 YSTCDFIM (thioxanthone ring between cysteine and methionine)

AIP2 GVNACSSLF (thioxanthone ring between cysteine and phenylalanine)

AIP3 INCDFLL (thioxanthone ring between cysteine and leucine-7)

AIP4 YSTCYFIM (thioxanthene ring between cysteine and methionine)

Abbreviations: A-alanine (Ala), C—cysteine (Cys), D—aspartic acid (Asp), E—glutamic acid (Glu), F—phenylalanine (Phe), G—glycine (Gly), H—histidine (His), I—isoleucine (Ile), K—lysine (Lys), L—leucine, (Leu), M—methionine (Met), N—asparagine (Asn), P—proline (Pro), Q—glutamine (Gln), R—arginine (Arg), S—serine (Ser), T—threonine (Thr), V—valine (Val), W—tryptophan (Trp), Y—tyrosine (Tyr).

AIP molecules are secreted outside bacterial cells with the participation of the SpsB peptidase, and the number of AIPs is directly proportional to the number of bacterial cells. Upon reaching a critical concentration, AIP binds to membrane histidine kinase (a product of the *agrC* gene) and activates it, causing histidine kinase to phosphorylate AgrA. The *S. aureus* cell produces only one type of AIP, and only this type of AIP activates histidine kinase, while other types of *S. aureus* AIP and quorum quenching (QQ) or quorum sensing inhibitor (QSI), produced mainly by other staphylococcal species, cause its repression [150,229]. The lifetime of AIP molecules is relatively short due to the rapid breakdown of the thiolactone ring. AIPs from groups 1 and 2 are exported by AgrB and cleaved at the N-terminus by the MroQ protease located in the membrane [150]. Phosphorylated AgrA protein (AgrA-P) activates and has high affinity for the *agr* P2, *agr* P3, and *psmalfa* and *psmbeta* promoters [231]. Activation of *agr* P2 facilitates continuous transcription of *agrA-D* to produce extracellular AIP. AgrA-P also binds to the P3 promoter to initiate transcription of RNAIII, which consists of 514 nucleotides (between nucleotides 70 and 165, it contains the *hld* gene transcript). The 5′ domain contains sequences complementary to the leader portion of transcripts of other genes (e.g., alpha hemolysin). The attachment of RNAIII to the transcript of another gene causes the paired strand of the transcript to bend and allows the ribosome to access the Shine-Dalgarno sequence (RBS) of that transcript, enabling its translation. The 3′ domain of RNAIII also has sequences complementary to the transcripts of certain genes (e.g., the *spa* gene). The attachment of RNAIII with its 3′ end to the transcript prevents ribosome access to the RBS and inhibits translation [7,230,231,232].

RNAIII regulates the production of many virulence factors either directly or indirectly by activating or inhibiting other regulatory systems.

The Agr system shows up-regulation of the genes of many proteins, including *hla*, *hld*, *hlgB*, *hlgC*, *psm beta 2*, *tst*, *aur*, *clfB*, *geh*, *lip*, *pls*, *splA*, *splB*, *splC*, *splD*, *splE*, *splF*, *sspA*, *sspB*, *sspC*, and *scpA*, and regulatory systems SaePQ, ArlRS, PhoP, WalK, ClpP, SarX, SigB, MgrA, and ESS (a gene cluster that encodes transport proteins and secretory proteins) [11,104,233].

The Agr system causes downregulation of most surface protein genes, *spa*, certain systems, and regulatory genes, e.g., srrAB [11,233].

The Agr system is activated by: MgrA (*agr* P3), CcpA (*agr* P3), SarA (*agr* P2), SarU (*agr* P2 and *agr* P3), and SarZ. The Agr system is inhibited by: ArlRS (*agr* P2 and *agr* P3), SrrAB (*agr* P2 and *agr* P3), sigma B (*agr* P3), CodY (*agrC*), Rsr (*agr* P3), SarR *agr* P2), SarT (*agr* P3), and SarX (*agr* P2 and *agr* P3) [234].

The activity of the global Agr (accessory gene regulator, quorum sensing system)/SarA (staphylococcal accessory regulator A) system, and the high activity of the SaePQRS (*Staphylococcus aureus* exoprotein expression response regulator) system, cause the synthesis of hemolysins, leukotoxins, PSM toxins (PSM alpha1-4, PSM alpha1-4), proteases, lipases, and proteins protecting *S. aureus* cells from the immune system (Sbi, Efb, Scn, Chp), and are more commonly observed in acute infections (invasive phenotype) caused by CA-MRSA. The activity of the Agr system significantly increases virulence and plays a particularly important role in skin infections, pneumonia, and endocarditis [48]. The lack of Agr system activity leads to the synthesis of adhesive proteins and biofilm formation (adhesive phenotype, chronic infections) and is characteristic of many HA-MRSA strains [48,235,236,237,238,239].

ArlRS (autolysis-related locus)

ArlRS (GenBank acc. AP017922; st. JP080, ST228). ArlS (signal transduction histidine-protein kinase ArlS; 451 aa). ArlR (response regulator ArlR; 219 aa) is stimulated by Agr. It directly or indirectly inhibits the expression of regulatory genes: *agr*RNAII, *agr*RNAIII (*agr* P2 and P3), *atlR*, *pmtR* (GntR family regulatory protein), *norG* (GntR family transcriptional regulator), *sarV*, and *sarY*; toxin genes: *hla, psm beta 1*, and *psm beta 2*; enzymes: *coa* and *lip*; surface protein genes: *ebh*, *spa*, *sasC*, *sasG*, *sdrD,* and *sdrE;* and superantigens like the protein *sll12* gene. It stimulates directly or indirectly inhibits the expression of regulatory genes: *mgrA*, *sarA*, *spx*, *lrgA* (murein hydrolase regulator LrgA), *nreA* (oxygen-sensing dissimilatory nitrate and nitrite reduction regulatory protein NreA), and *nreC* (DegU family transcriptional regulator); toxin genes: *lukFS*, *lukAB*, *hlb*, *hlgB*, *hlgC*, and *selX*; surface protein genes: *sbi*, *eap*, *scn*, and *chp*, capsule gene *cap5*, and enzyme genes: *sak*, *lip*, *gehB*, and *nuc* [240,241,242,243].

SaeRS (saePQRS, *S. aureus* exoprotein expression)

The system includes: SaeRS (GenBank acc. AP017922; st. JP080, ST228), SaeR (response regulator *saeR;* 228 aa), and SaeS (histidine protein kinase *saeS*, 351 aa). SaeQ and SaeP inhibit the expression of *saeS.* The *saeS* phosphorylates SaeR, which induces the production of exoproteins, including many virulence factors. It is essential for the virulence of skin infections and pneumonia. SaeRS causes upregulation of *coa*, *nuc*, *fnbA*, *fnbB*, *efb*, *set15*, *hla*, *hlb*, *hlgC*, and *tst*, and downregulation of *agrA*. It is inhibited by CodY and sRNA and activated by sigma B [48,234,244,245,246,247].

SrrAB (staphylococcal respiratory response AB)

SrrAB (GenBank acc. AP017922; st. JP080, ST228) includes *srrA* (*istB*-like ATP-binding protein, 52 aa) and *srrB* (sensor protein SrrB, 583 aa). SrrAB activates the expression of *plc* genes *ica* operon and *agr*RNAIII. It inhibits the expression of *agr* P2 and *agr* P3, *tst*, and *spa*. It participates in the adaptation of *S. aureus* to anaerobic growth [48,248,249].

WalRK (YycGF)

WalRK (YycGF) is a regulator of cell wall metabolism in *S. aureus*. Upon activation by the WalK sensor histidine kinase, the WalR response regulator binds to the promoter sequence and increases the expression of staphylococcal autolysins, e.g., atlA, *sle1*, and *lytM*. It stimulates the SaeRS regulatory system. Point mutations in the *walRK* operon are frequently reported in VISA and daptomycin-resistant *S. aureus* [250]. WalRK (YycG/YycF) regulates the expression of SdrD, EbpS, Sak, and LytM (peptidoglycan hydrolase) [251,252,253].

GraRS

GraRS positively regulates the expression of the *dlt* operon, whose products play an important role in evading host defense mechanisms such as cationic antimicrobial peptides and neutrophil killing. In addition, GraRS activity is necessary for the expression of VISA-type vancomycin resistance in the Mu50 strain [254,255].

YvqF/VraSR

YvqF/VraSR is a two-component sensory regulation system involved in the control of bacterial cell wall metabolism. In addition, *yvqF* negatively regulates *vraSR*, which is responsible for VISA-type vancomycin resistance [255].

LuxS/AI-2 QS

LuxS/AI-2 QS is activated by the QS signal. It inhibits PIA-dependent biofilm formation and Rbf regulator expression [70].

KdpD/KdpE

KdpD/KdpE activates *spa* and *cap*. It inhibits *hla*, *hlgB*, *geh*, and *aur*. It is activated by LuxS/AI-2 QS and by low potassium (K^+^) concentration. It is inhibited by Rot [256].

FakAB1B2 (Fatty acid kinase)

FakB can probably transfer a phosphoryl group (acyl-PO4) to SaeR, leading to the activation of the SaeRS regulatory system. Mutations in the *fakA*, *fakB1*, and *fakB2* genes result in the lack of alpha toxin production [257].

ClpXP

ClpXP positively controls the translation of the Rot regulatory protein. It downregulates alpha-hemolysin, gamma-hemolysin, leukotoxins, and *splA-splF* serine proteases [258].

SpoVG (global stress response regulator)

SpoVG positively regulates the cap operon responsible for capsule synthesis. It activates *icaR* expression, which consequently inhibits PIA-dependent biofilm formation [103].

### 9.2. Regulatory Transcription Factors

Rsr (repressor of sarR)

Rsr inhibits *sarR* and *agr P3* [259].

CcpA (catabolite control protein A)

Activates Agr (*agr* P3), and indirectly *hla* and *spa*, both in the presence and absence of glucose. It affects resistance to oxacillin and glycopeptides [260].

Fur (ferric uptake regulator)

Fur is a repressor of transcription of many genes. The lack of Fur repression in the case of iron starvation directly or indirectly activates the synthesis of many virulence factors: hemolysins, hydrolases, cytolysins, nucleases, proteases and lipases, chemotaxis inhibitory protein (CHIP), staphylococcal complement inhibitor (SCIN), coagulase, exotoxins Ssl 1, Ssl 2, Ssl 6, Ssl 7, Ssl 9, and Ssl 11, biofilm-forming proteins Eap and Emp, and operons responsible for iron uptake [209,211].

Rbf (global regulators of biofilm formation from the AraC/XylS family)

Rbf activates *sarX* and, through it, inhibits the expression of the *icaR* gene, promoting PIA-dependent biofilm synthesis [70]. It inhibits the expression of Hla and PSM alpha [108,261].

Rsp (regulator from the AraC/XylS family)

Rsp inhibits biofilm formation at the primary aggregation stage by repressing *fnbA*, which involves binding to the *fnbA* gene promoter. Rsp inhibits 75 genes, including *fnbA*, *fnbB*, *clfA*, *spa*, *sdrD*, *sdrE*, *sasG*, *sel*, and the MarR regulator. Rsp activates the expression of 22 genes, including *lukD*, *hlb*, *geh*, *splA*, *splB*, *splC*, and *plc* [108].

SRR42 (non-coding transcript; sRNA)

SRR42 is induced by components of the host immune system, inhibited by the PerR (peroxide repressor) regulator; it stimulates the translation of LukAB, LukSF, Hla, HlgA, HlgB, Sbi, Efb, Sak, and Spl (serine protease) and activates SigS (sigma factor S), SarZ, SarR, and MntR (metalloregulator). It inhibits the production of Aur (aureolysin), SspA (V8 protease), SraP, and IsaB proteins, and inhibits the metalloregulator Fur [262].

Rot (toxin repressor)

Rot activates *sarS*. It inhibits the expression of *hla*, *hlb*, *tst*, *sec*, *sed*, *spa*, serine and cysteine proteases, and lipase. Rot can bind to the P1 promoter of the *sae* locus and inhibit its activity. It is inhibited by Agr. Rot translation is positively regulated by ClpXP [205,231,263,264].

MgrA (other names NorR, Rat, transcriptional regulator MgrA)

MgrA activates *agr* P3, *cap5*, *cap8*, *nuc*, *lytSR*, *arlSR*, and *sarS;* it inhibits *hla*, *spa*, *coa*, *sspA*, *norA*, and *sarV*. It is activated by *agr*RNAIII [86,265].

SigB (sigma B)

Sigma factors bind to RNA polymerase, ensuring the specificity of the target gene during the transcription initiation process. Sigma factor 70 is responsible for gene expression during exponential bacterial growth, after which other sigma factors take over regulatory functions, e.g., sigma B, which is responsible for the expression of approximately 200–250 genes, including many virulence genes. SigB can be activated by the Agr system or act independently of the Agr system. SigB activates *sarA* P3 and *sarS*. It inhibits *agr* P3 [11,266,267].

Spx (transcriptional regulator involved in the response to oxidative stress)

Spx is activated by ArlRS. It activates the expression of *icaR*, thereby inhibiting PIA-dependent biofilm synthesis [240].

TcaR (TcaR transcription regulator)

The TcaR protein has 151 aa (GenBank acc. AP017922). It inhibits *icaADBC*. It activates *sarS* [231].

CodY (global transcriptional regulator CodY)

The CodY protein binds to the AATTTTCWGAAAATT motif of chromosomal DNA. CodY is a repressor of the *sae*P1 promoter. It inhibits protease production, PIA-dependent biofilm production, and the expression of *agrC* and the RsaD regulator. It stimulates biofilm formation with eDNA during mild acid stress [231].

RsaD (sRNA)

RsaD is responsible for regulating cell death during mild acid stress. It is inhibited by CodY [268].

ScrA (S. aureus clumping regulator A)

ScrA is activated by ArlRS and indirectly by MgrA. It activates SaeRS and the expression of surface adhesins. It inhibits the release of secretory proteases. The expression of ScrA is inhibited by feedback from the activated SaeRS system [269].

SarA (staphylococcal accessory regulator A)

SarA is transcribed from three promoters: P1, P2, and P3. Each transcript contains the sarA gene. It inhibits the transcription of *sarT, sarS, sarV, rot,* and, among others: *spa*, *atl*, *aur*, *lip*, *nuc*, *sspB,* and *sspC*. It increases the expression of *cap*, *agr P2*, *hla*, *hld*, *hlgB*, *hlgC*, *pls*, *set9*, *splA*, *splB*, *splC*, *splF*, *psm beta 2*, *fnbA*, *fnbB*, and *cna*, among others. It regulates the expression of RNAII and RNAIII in the Agr system. The sarA regulator is activated by *sarS, sarT, rot, mgrA, tcaR* and *sigB* [233,270,271,272].

SarR (staphylococcal accessory regulator R)

SarR inhibits transcription of *sarA P1*, *sarA P2*, *and sarA P3*, *agr P2*, *hla*, *hlb*, and *spa* [231,273].

SarS (sarH1, staphylococcal accessory regulator S)

SarS inhibits transcription of *hla* and activates spa. It is activated by SigB, SarT, MgrA, TcaR, and Rot [270].

SarT (staphylococcal accessory regulator T)

SarT inhibits transcription of *agr P3, hla,* and *sarU*. It activates *sarS* and is inhibited by SarA and *agr*RNAIII [274].

SarU (staphylococcal accessory regulator U)

SarU activates transcription of *agr* P2 and P3, *sarV, ccpA*. It is inhibited by *SarT* [231,275].

SarV (staphylococcal accessory regulator V)

SarV regulates the expression of autolysin genes. It activates murein hydrolase [276].

SarX (staphylococcal accessory regulator X)

SarX is activated by Agr and regulator Rbf. It inhibits the expression of *agr* P2 and *agr* P3 and the *icaR* gene, promoting PIA-dependent biofilm synthesis [70].

SarZ (staphylococcal accessory regulator Z)

SarZ is activated by regulator SRR42. It activates Agr [262].

## 10. Genetic Localization of Virulence Factors

Most virulence genes are located on the bacterial chromosome. Certain regions of the chromosome are called genomic islands (vSa). Genomic islands are located within a specific chromosome locus associated with intact or reduced DNA recombinase. Three such genomic islands are identified in *S. aureus* genomes: vSAalpha, vSAbeta, and vSagama. Within vSAalpha, there are many genes referred to as superantigen-like or enterotoxin-like (*ssl*, *sel*) and lipoprotein (*lpl*) genes. The vSAbeta island may contain clusters of *spl* (serine protease-like) and enterotoxin (*egc*) genes, the lantibiotics/bacteriocins biosynthesis operon (*bsa*), the hyaluronidase precursor gene (*hysA*), and genes encoding two-component leukocidin (*lukED*). The genomic *vSA* and chromosomal egc (enterotoxin gene cluster) islands that are not part of SaPI or plasmids are not classified as mobile elements. Several egc have been described, e.g., egc1 contains the genes *seg*, *sen*, *sei*, *sem*, *seo* (GenBank acc. EU885495); egc2 contains the genes *seg*, *sen*, *selu*, *sei*, *sem*, *seo*; egc3 contains the genes *seg*, *sen*, *selu*, *sei*, *sem*, *seo* (GenBank acc. EU885494, EU885493, EU885496); egc4 contains the genes *seg*, *sen*, *selw*, *selv*, *seo;* and *egc* with a size of 26,263 bp (GenBank acc. MN450304) contains the genes *sel27*, *sel28*, *seg*, *sen*, *selu2*, *sei*, *sem*, *seo* [277,278,279]. In addition, mobile elements such as SaPI (pathogenicity island), prophages (phi), transposons (Tn), SCCmec cassettes, and plasmids outside the chromosome may be present in the chromosome. The most important plasmids, pathogenicity islands, prophages, and enterotoxin gene clusters encoding virulence factors are presented in Table 7 according to previous studies [10,11,23,24,28,37,39,40,41,42,93,94,136,158,159,161,162,183,184,185,186,188,189,190,277,280,281,282,283,284].

## 11. The Importance of Pathogenic Factors in Skin Diseases

*S. aureus* and/or the pathogenic factors it produces can cause skin and subcutaneous tissue diseases such as boils, furunculosis and carbuncles, folliculitis, staphylococcal impetigo including bullous impetigo, cellulitis, staphylococcal erysipelas, and wound infections, as well as staphylococcal necrotizing fasciitis and staphylococcal scalded skin syndrome (SSSS). The factors enabling colonization of the skin and mucous membranes at the onset of infection are described in Chapter 2. It has been shown that the severity and course of skin and soft tissue infections may be associated, among other things, with the production by *S. aureus* of virulence factors such as PVL, alpha-hemolysins, beta-hemolysins, leukocidin (lukED), PSM, and exfoliatins. Their role is discussed in more detail in Chapter 6—Toxins. It has been shown that patients infected with PVL-producing strains more often required surgical intervention (abscess incision), while those infected with non-PVL-producing strains more often required antibiotic therapy [129,285]. Surface proteins such as coagulases, Sbi, and Spa also participate in the formation of staphylococcal abscesses. The above diseases should be distinguished from the action of *S. aureus* as one of many contributing factors causing symptoms or affecting the course of certain skin diseases. *S. aureus* rarely colonizes the skin of healthy individuals because most *S. aureus* strains (except for some CA-Sa USA300 strains with the ACME element and SpeG protein) are not adapted to do so. In addition, coagulase-negative staphylococci (CoNS), which dominate the skin flora, secrete lantibiotics, antimicrobial peptides, and quorum quenching (QQ) or quorum sensing inhibitor (QSI) molecules that can inhibit QS signals in *S. aureus*. Chronic inflammation, elevated skin pH, and a damaged epidermal barrier including filaggrin deficiency facilitate *S. aureus* colonization. It has also been demonstrated that mutations in the gene encoding human TLR-2 lead to increased colonization by *S. aureus* and an increased risk of atopic dermatitis (AD). Increased colonization of *S. aureus* has been found in AD, but also in some other skin diseases like skin lymphomas, and to some extent, even psoriasis. The adverse effect on the course of the disease is best demonstrated in the case of AD. The density of the bacteria is correlated with the onset of disease [150]. Genomic differences between *S. aureus* strains associated with AD and healthy skin were demonstrated based on whole genome sequencing. In colonized infants who did not develop AD at 1 year of age, *S. aureus* mutations causing loss of Agr system function were found, while *S. aureus* isolated from infants with AD had an active Agr system [286]. Toxins and enzymes activated by the Agr system, such as PSM alpha, delta toxin, alpha-hemolysin (Hla), lipoteichoic acid (LTA), peptidoglycan (PGN), and Spa, are involved in skin inflammatory reactions. The delta toxin causes mast cell degranulation and induces type II inflammation, while PSM alpha causes the release of IL-1alpha and IL-36 alpha alarmins from keratinocytes and induces IL17-mediated skin inflammation. LTA and PGN induce inflammatory reactions in the skin as Toll-like receptor ligands [150,287,288,289]. Polyclonal lymphocyte activation and uncontrolled inflammatory response may also be caused by staphylococcal superantigens such as TSST-1 or enterotoxins. *S. aureus* producing TSST-1 is significantly more common in patients with severe HSV infection, ADEH—atopic dermatitis eczema herpeticum—than in patients with AD alone. In addition, *S. aureus* produces proteases that directly damage the epidermal barrier. It has also been shown that the bacterium activates the production of serine proteases and metalloproteinases by fibroblasts in the host’s skin [290]. On the other hand, it has been shown that topical treatment with corticosteroids, calcineurin inhibitors, and systemic dupilumab, despite their immunosuppressive effects, causes a rapid decrease in the number of *S. aureus* on the skin in patients with AD [291].

It is suspected that SE-producing *S. aureus* plays a role in the pathogenesis of cutaneous T cell lymphomas (CTCL). It has also been demonstrated that infection with SE-producing *S. aureus* can cause malignant T cells to become resistant to chemotherapy. SE-producing *S. aureus* and a recombinant SE can significantly inhibit cell death induced by the HDAC inhibitor romidepsin in T cells from patients with SS. Being superantigens, SEs bind to malignant lymphocyte TCR, causing activation of lymphocyte cell-specific protein-tyrosine kinase (LCK) and the metabolic pathway associated with NF kappa beta. Furthermore, parallel activation of non-malignant T lymphocytes leads to the production of cytokines from the IL-2 group, which act on malignant lymphocytes, leading to the activation of JAK-STAT metabolic pathways. Both above mechanisms may be responsible for resistance to chemotherapy [292].

In patients with psoriasis, the skin microbiome is more diverse and *S. aureus* colonization is less common than in patients with AD. However, in the study group of patients with psoriatic erythroderma, *S. aureus* colonization was significantly increased. Patients were found to have reduced concentrations of antimicrobial peptides such as cathelicidin (LL-37), human β-defensin 2 (hBD2), and ribonuclease 7, which may have promoted staphylococcal colonization. Similarly to patients with AD, but unlike patients with psoriasis vulgaris, patients with PE also had elevated IL4 and 13 levels. *S. aureus* colonization could be associated not only with the maintenance of inflammation but also with an increased risk of systemic infections that are dangerous for these patients [293].

*S. aureus* infections (often MRSA), along with other bacteria, are also common in patients with hidradenitis suppurativa and influence the course of the disease and the maintenance of inflammation. The production of adhesins, biofilm formation, and the production of toxins, including SE and hemolysins, may all play a role. A study conducted in a group of 39 patients with HE suggests a poorer response to adalimumab treatment in patients colonized with *S. aureus*. However, as the authors themselves suggest, this finding requires confirmation in a larger group [294,295].

## 12. Conclusions

In summary, *S. aureus*, evolving under the influence of diverse environmental conditions, has developed many different mechanisms that allow it to survive and expand. The course of a specific infection depends not only on the presence of the given pathogenicity genes, but also on the action of numerous systems regulating the expression of these genes. These systems are often able to sense and respond to certain environmental variables, such as oxidative stress, the presence of iron in the environment, or the number/density of surrounding bacterial cells. This system of interactions makes *S. aureus* one of the most adaptable pathogens.

## Figures and Tables

**Table 1:** Complement-inhibiting proteins.

Complement-Inhibiting Protein	Target Protein	Mechanism of Action
Efb, Ecb	C3b (C3d domain), FH	A change in the conformation of C3b, hindering its binding to factor B, and the formation of active C3 convertase enhances the regulatory effect of FH.
SCIN	C3bBb convertase	Stabilization of the protein in inactive form, preventing the production of C3a, C3b, and C5a.
Sbi	C3 C3b FH	Several mechanisms: (1) Binds to C3, leading to the activation of an alternative complement pathway and the consumption of this component. (2) Stabilizes C3d dimers. (3) Forms complexes with C3b and FH, causing a reduction in complement activation.

**Table 2:** Proteins protecting bacteria from the immune system.

Protein	Gene	Protein (aa)	Genetic Element	GenBank acc.
Spa	*spa*	516 454	Chr: st. MRSA252 Chr: st. JP1	BX571856 JQ066313
Sbi	*sbi*	437	Chr: st. JP080	AP017922
Chp (CHIPS)	*chp*	149 149	phiSA3 in *hlb*; chr: st. MRSA252 Chr: st. RUH2 (MRSA, CC15)	BX571856 MF185201
Scn (SCIN)	*scn*	116 116 124 124	SaPIpig2; chr: st.ST398 phi Sa3 in *hlb*; chr: st. OC8 SaPIbov4 chr: st. BA4 SaPIbov5 chr: st. JP5338	MW589253 LC129040 HM211303 ADN53657
eqSCIN	*scn*	116	phi Saeq1; chr: st. 3711	LT671578
Vwb	*vwb*	499 499 499	SaPIpig2; chr: st.ST398 SaPIbov4 chr: st. BA4 SaPIbov5 st. JP5338	MW589253 HM211303 ADN53657
Ecb	*ecb*	109	Chr: st. R8015	CP156750

Abbreviations: Chr:, chromosome; st., strain; aa, number of amino acids; SaPI, *S. aureus* pathogenicity island; phi, prophage.

**Table 3:** Human specificity TCR Vbeta for staphylococcal superantigens.

Toxin	Human Specificity TCR Vbeta
TSST-1	2, 12-3, 12-4
SEA	1, 1,1, 5, 5.2, 5.3, 6, 6,3, 6.4, 6.9, 7, 7.2, 7.3, 7.4, 9, 9.1, 15, 16, 18, 21, 22, 24
SEB	1, 3, 6, 8, 12, 13.2, 14, 15, 17, 20
SEC	3, 12, 13.2, 14, 15, 17, 20
SEC-1	3, 12, 13.2, 14, 15, 17, 20
SEC-2	3, 12, 13,1, 13.2, 14, 15, 17, 20
SEC-3	3, 12, 13,1, 13.2, 14, 15, 17, 20
SED	1, 3, 5, 5.1, 5.2, 5.3, 8, 9, 12, 14
SEE	5, 5.1, 6, 6.3, 6.4, 6.9, 8, 8.1, 9, 13.1, 16, 18, 21, 21.3
SEG	3, 12, 13, 13.1, 13.2, 14, 15
SEH	Valpha8, Valpha10
SEI	1, 5, 5.1, 5.2, 5.3, 6, 23
SElJ	8, 21, 21.3
SEK	1, 5, 5.1, 5.2, 6, 6.7
SEL	1, 5, 5.1, 5.2, 5.3, 6.7, 7, 7.1, 16, 22, 23
SEM	8, 9, 18, 21, 21.3
SEN	5.1, 7, 8, 9, 17
SEO	5, 6, 7, 7.1, 8, 9, 18, 21.3
SEP	5, 5.1, 8, 16, 18, 21, 21.3
SEQ	2, 5.1, 6, 6.7, 21, 21.3
SER	3, 11, 12, 13.2, 14
SES	9, 16
SElU	13.2, 14
SElV	6, 6.7, 18, 21, 21.3
SElX	1, 6, 18, 21

**Table 4:** The most important *S. aureus* surface proteins and their selected targets.

Protein	Gene	Protein (aa)	Genetic Element	GenBank acc.	Selected Targets
Bap	*bap*	2276	SaPIbov2; Chr: st. V329	AY220730	Endoprosthesis polystyrene, mammary gland epithelium(animal)
ClfA	*clfA*	1029	Chr: st.MRSA252	BX571856	Fibrinogen
ClfB	*clfB*	873 1017	Chr: st. MRSA252 Chr: st.JP080	BX571856 AP017922	Fibrinogen, Epidermis, nasal epithelium
FnbA	*fnbA*	1018	Chr: st. MRSA252	BX571856	Fibrinogen, fibronectin, elastin
FnbB	*fnbB*	940	Chr: st. MRSA252	BX571856	Fibrinogen, fibronectin, elastin
Cna	*cna*	1183	Chr: st. MRSA252	BX571856	Collagen
SasG	*sasG*	987	Chr: st. MR23	LC388387	Epidermis, nasal epithelium
Pls	*pls*	1637	Chr: st.1061	AF115379	Glycolipids, nasal epithelium
IsdA	*isdA*	354	Chr: st.Jp080	AP017922	Fibrinogen, fibronectin, nasal epithelium
Emp	*emp*	340	Chr: st. KAM440	AP040133	Fibrinogen(nc) fibronectin(nc) vitronectin(nc)
Eap	*eap*	584	Chr: st.MR23	LC388386	Fibrinogen(nc) fibronectin(nc) vitronectin(nc)
Atl	*atl*	1246	Chr: st. NTUH9148	KT894182	Fibrinogen(nc) fibronectin(nc) vitronectin(nc) Endoprosthesis polystyrene (nc)
Bbp	*bbp*	1087	Chr: st. YM514	KY095832	Bone sialoprotein
Aaa	*aaa*	335	Chr: st. DSM 110898	CP192215	Fibrinogen(nc) fibronectin(nc) vitronectin(nc)
SdrC	*sdrC*	947	Chr: st. 45030564 Chr: st. NRS384; USA300	AM076176 CP027476	Bone sialoprotein, nasal epithelium
SdrD	*sdrD*	1381	Chr: st. NRS384; USA300	CP027476	Bone sialoprotein, nasal epithelium
SdrE	*sdrE*	1154	Chr: st. NRS384; USA300	CP027476	Fibrinogen bone sialoprotein

Abbreviations: Chr:, chromosome; st., strain; aa, number of amino acids; SaPI, *S. aureus* pathogenicity island, nc, non covalently.

**Table 5:** Pore-forming toxins, exfoliatins, and exotoxins.

Protein	Gene	Protein (aa)	Genetic Element	GenBank acc.
Hla	*hla*	319	Chr: st. MRSA252	BX571856
Hlb	*hlb*	330	Chr: st. MRSA252; CDS is disrupted by the integration of the phiSa3(252) after residue 61	BX571856
Psm alpha 1	*psm alpha 1*	21	Chr: st. JP1	JQ066321
Psm alpha 2	*psm alpha 2*	21	Chr: st. JP1	JQ066321
Psm alpha 3	*psm alpha 3*	22	Chr: st. JP1	JQ066321
Psm alpha 4	*psm alpha 4*	20	Chr: st. JP1	JQ066321
Psm-mec	*psm-mec*	22	SCCmecIIIR st.16K (ST239)	AB539727
Psm beta 1	*psmB1*	44	Chr: st. JP080	AP017922
Psm beta 2	*psmB2*	44	Chr: st. ST20130941	CP012978
Hld	*hld*	45	Chr: st. JP080	AP017922
HlgA (S)	*hlgA (hlg1)*	309	Chr: st. MRSA252	BX571856
HlgB (F) LukF-R (F)	*hlgB (hlg2)* *lukF-R*	325 325	Chr: st. MRSA252 Chr st. P83	BX571856 X64389
HlgC (S) LukS-R (S) Luk-S (S)	*hlgC (hlg3)* *lukS-R* *luk-S*	315 315 315	Chr: st. MRSA252 Chr: st. P83 Chr: st. MRSA no 4	BX571856 X64389 S65052
LukS-PV (S)	*lukS-PV*	312 312 312	Chr: st. HT20060855 phi in chr: st.1_9; CC22 phi Sa2/chr: USA300 st.NRS384	EU518770 HM584702 CP027476
LukF-PV (F)	*lukF-PV*	323 325 325	Chr: st. HT20060855 phi in chr: st. 1_9; CC22 phi Sa2/chr: USA300 st.NRS384	EU518770 HM584702 CP027476
LukD (F)	*lukD*	327 327	SaPIn3; chr: st. N315 Chr: st. Newman	BA000018 Y13225
LukE (S)	*lukE*	311 314 311	SaPIn3; chr: st. N315 Chr: st. Newman Chr: USA300 st.NRS384	BA000018 Y13225 CP027476
LukF (F)	*luk-F*	323 327	Chr: st. MRSA no 4 Chr: USA300 st.NRS384	S65052 CP027476
LukG (LukA)	*lukG (lukA)*	338	Chr: st. KAM440	AP040133
LukH (LukB)	*lukH (lukB)*	350	Chr: st. KAM440	AP040133
LukQ (F) *	*lukQ*	326	phiSaeq1 chr: st. 3711	LT671578
LukP (S) *	*lukP*	311	phiSaeq1 chr: st. 3711	LT671578
Eta	*eta*	311	SaPI2R	AB983196
Etb	*etb*	277	p (unnamed) st. TY4	NC_003265
Etb	*etb*	281	Chr: st. JP080	AP017922
Exotoxin 6	*set6*	226	SaPIn2 st. N315	BA000018
Exotoxin 7	*set7*	231	SaPIn2 st. N315	BA000018
Exotoxin 8	*set8*	356	SaPIn2 st. N315	BA000018
Exotoxin 9	*set9*	292	SaPIn2 st. N315	BA000018
Exotoxin 10	*set10*	234	SaPIn2 st. N315	BA000018
Exotoxin 11	*set11*	231	SaPIn2 st. N315	BA000018
Exotoxin 12	*set12*	232	SaPIn2 st. N315	BA000018
Exotoxin 13	*set13*	232	SaPIn2 st. N315	BA000018
Exotoxin 14	*set14*	227	SaPIn2 st. N315	BA000018
Exotoxin 15	*set15*	227	SaPIn2 st. N315	BA000018

Abbreviations: chr, chromosome; st., strain; aa, number of amino acids; SaPI, *S. aureus* pathogenicity islands; phi, prophage; * isolated from animal biotypes of *S. aureus.*

**Table 6:** TSST-1 and enterotoxins.

Protein	Gene	Protein (aa)	Genetic Element	GenBank acc.
TSST-1	*tst*	234	SaPISaitama2 st. Saitama2 SaPIj st. SI1; CA-MRSA SaPIn1 st. N315 SaPI2 st. RN3984 SaPI2R st. OC3 SaPI68111 st.68111 SaPIj50 st. NN50 (CA-MRSA;ST8)	LC315809 LC706631 BA000018 EF010993 AB983196 JN689383 AB679717
SEA	*sea*	257	Chr: st. KAM440 Chr st. V8 Chr st. JKD6004-DR Chr st. Sa230502_barcode25 phi Sa3(252) st.MRSA252	AP040133 CP079715 CP040625 CP168017 BX571856
SEB	*seb*	266	SaPI3 st. COL SaPIivm60 st.IVM60 SaPI st.IVM10 Chr: st. KAM440	AF410775 AB704539 AB716349 AP040133
SEC	*sec*	266	SaPISaitama2 st. Saitama2 SaPI st. 356P SaPITokyo12381 st. Tokyo12381	LC315809 MN450305 AB860418
SEC1	*sec1*	259	Chr: st. DAR4145	CP010526
SEC2	*sec2*	266	Chr st. DAR4145 Chr: st. Sa230905_barcode06	CP010526 CP168012
SEC3	*sec3*	266 266 212 266	SaPIj st. SI1; CA-MRSA SaPIn1 st. N315 SaPI68111 st.68111 SaPIj50 stNN50 (CA-MRSA;ST8)	LC706631 BA000018 JN689383 AB679717
SED	*sed*	258	pER10678.3A.1 st. ER10678.3 pIB485 genomic DNA	CP051928 M94872
SEE	*see*	260	Chr: st. 1563 (partial)	AJBV01000012
SEG	*seg*	258	Chr st. sa230502_ barcode25 Chr: st. sa230905_barcode06 SaPI genomic DNA SaPIn3; chr: st. N315 egc chr: st.364P	CP168017 CP168012 MN450304 BA000018 MN450304
SEH	*seh*	241	Chr: st. MISS6027 (partial) Chr: st. KAM440	JBPH01000006 AP040133
SEI	*sei*	242	Chr: st. sa230905_barcode06 Chr: st. sa230905_barcode06 Chr st. sa230502_ barcode25 SaPI genomic DNA SaPIn3; chr: st. N315 egc chr: st.364P	CP168012 CP168012 CP168017 MN450304 BA000018 MN450304
SEJ	*sej*	268	pSa231003_barcode77A st. sa231003_barcode77 pER10678.3A.1 st. ER10678.3 pIB485 st. KSI1410 pF5 st. Fukuoka5 pH3 st.Hiroshima3	CP168011 CP051928 AF053140 AB765928 NZ_AB765929
SEK	*sek*	242	SaPI3 st.COL SaPIj11 st. J11 Chr: st. KAM440 SaPI1 st. OC3 SaPI5 st. USA300_FPR3757	AF410775 AB704541 AP040133 AB983198 CP000255
SEL	*sel*	240	Chr: st. sa230905_barcode06 SaPI st. 356P SaPISaitama2 st.Saitama2 SaPIj st. SI1; CA-MRSA SaPIn1 st. N315 SaPI68111 st. 68111 SaPIj50 st.NN50 (CA-MRSA;ST8)	CP168012 MN450305 LC315809 LC706631 BA000018 JN689383 AB679717
SEM	*sem*	239	Chr: st. sa230905_barcode06 Chr: st. sa230502_ barcode25 egc chr: st. 364P SaPIn3; chr: st. N315	CP168012 CP168017 MN450304 BA000018
SEN	*sen*	251 251 251 251 258	Chr: st. pt236 Chr: st. sa230905_barcode06 Chr: st. sa230502_ barcode25 egc chr: st. 364P SaPIn3; chr: st. N315	CP049472 CP168012 CP168017 MN450304 BA000018
SEO SEO	*seo*	254 260 260 260	Chr: st. BSAR207 Chr: st. sa230905_barcode06 egc chr: st. 364P SaPIn3 chr: st. N315	CSPC01000129 CP168012 MN450304 BA000018
SEP	*sep*	257 260	Chr: st. DAR4145 Chr: st. sa230905_barcode06	CP010526 CP168012
SEQ	*seq*	242 242 244 256 242	SaPI3 st. COL Chr: st. NAS_AN_102 SaPIj11 st. J11 SaPI1 st. OC3 SaPI5 st. USA300_FPR3757	AF410775 CP062448 AB704541 AB983198 CP000255
SER	*ser*	259	pF5 st. Fukuoka5 pH3; st. Hiroshima3	AB765928 NZ_AB765929
SES	*ses*	257	Chr: st. Hiroshima3 pF5 st. Fukuoka5 pH3; st. Hiroshima3	AB765929 AB765928 NZ_AB765929
SET	*set*	216	pF5 st. Fukuoka5 pH3; st. Hiroshima3	AB765928 NZ_AB765929
SEU	*seu*	261	Chr st. sa230502_ barcode25 Chr: st. sa230905_barcode06	CP168017 CP168012
SElU2	*sel2*	256	egc chr st.364P	MN450304
SElW	*selw*	250	Chr: st.SC912 (ST45, CA-MRSA)	MN257146
SEX	*selx*	203	Chr: st. sa230905_barcode06 Chr: st. KAM440	CP168012 AP040133
SEY	*sey*	221	Chr: st. Dresden-27575	CP054876
SElZ	*selz*	259	Chr: st. SC732 (ST121, CA-MRSA)	MN257154
SEl26	*sel26*	250 234 247	Chr: st. TGD15 Chr: st. KAM440 Chr: st. SJTU F20365	CP098675 AP040133 MF370878
SEl27	*sel27*	250 241	Chr: st. SJTU F20365 egc chr: st. 364P	MF370878 MN450304
SEl28	*sel28*	250	egc chr: st. 364P	MN450304
SEl30	*sel30*	250	p2021-15363st.WA121-2021_15363 p2021-16354-2st.WA121-2021_16354 p2010-15611-2st.WA121-2010_15611 p (unnamed) st. E1 pWSI1st. SI1	NZ_CP093934 NZ_CP093937 NZ_CP093932 NC_010077 NZ_LC383633
SEl31	*sel31*	256	p1_96 st. t16335 pCC205_1 st. C205	NZ_JBLEAE010000002 CP127810
SEl32	*sel32*	243	p1_96 st. t16335 pC205_1 st. C205	NZ_JBLEAE010000002 CP127810
Yent1	*yent1*	133	SaPIn3; chr: st. N315	BA000018
Yent2	*yent2*	136	SaPIn3; chr: st. N315	BA000018
SEl6	*set6*	226	Chr: st. DAR4145	CP010526
SEl7	*set7*	231	Chr: st. DAR4145	CP010526
SEl8	*set8*	369	Chr: st. DAR4145	CP010526

Abbreviations: SE, enterotoxin; SEl, enterotoxin-like; chr, chromosome; st., strain; aa, number of amino acids; SaPI, *S. aureus* pathogenicity island; phi, prophage; egc, enterotoxin gene cluster; p, plasmid.

**Table 7:** Mobile elements containing virulence genes.

ELEMENT	SIZE (bp)	GenBank acc.	Virulence Genes
SCCmecIIIR	30,858	AB539727	*psm-mec*
SCCmecXIV	81,000	LC440647	*ACME II′*
pF5	43,265	AB765928	*sej*, *ser*, *ses*, *set*
pER10678.3A.1	27,273	CP051928	*sed*
pH3	31,888	NZ_AB765929	*ser*, *sej*, *set*, *ses*
pIB485	partial partial	M94872, AF053140	*sed*, *sej*, *ser*
p2021-15363	34,990	NZ_CP093934	*sel30*
p2021-16354-2	34,986	NZ_CP093937	*sel30*
p2010-15611-2	34,990	NZ_CP093932	*sel30*
p (unnamed)	34,986	NC_010077	*sel30*
pWSI1	32,573	NZ_LC383633	*sel30*
p1_96	50,738	NZ_JBLEAE010000002	*sel31*, *sel32*
pCC205_1	29,415	CP127810	*sel31*, *sel32*
p (unnamed) pETB	38,211 38,211	NC_003265 AP003088	*etb* *etb*
pEDINA	34,986	AP003089	*edin*
psa231003_barcode77A	27,268	CP168011	*sed*, *sej*, *ser*
SaPI1	14,577	AB983198	*sek*, *seq*
SaPI2	14,755	EF010993	*tst*
SaPI2R	14,819	AB983196	*eta*, *tst*
SaPI3	15,953	AF410775	*seb*, *sek*, *seq*
SaPI5	13,292	CP000255	*sek*, *seq*
SaPIj	15,405	LC706631	*tst*, *sec3*, *sel*
SaPIj11	15,751	AB704541	*selk*, *selq*
SaPIj50	15,206	AB679717	*tst*, *sec3*, *sel*
SaPIn1	15,113	BA000018	*sel*, *sec3*, *tst*
SaPIn2	29,342	BA000018	*set6*, *set7*, *set8*, *set9*, *set10*, *set11*, *set12*, *set13*, *set14*, *set15*
SaPIn3	26,255	BA000018	*lukD*, *lukE*, *seg*, *sen*, *yent2*, *yent1*, *sei*, *sem*, *seo*
SaPISaitama2	15,401	LC315809	*sec*, *sel*, *tst*
SaPI (fhuD)	15,756	AB983199	*fhuD*
SAPIpig2	13,505	MW589253	*scn*, *vwb*
SaPIivm60	15,391	AB704539	*seb*
SaPITokyo12381	15,419	AB860418	*sec*
SaPIbov2	27,031	AY220730	*bap*
SaPIbov4	13,950	HM211303	*vwb*, *scn*
SaPIbov5	13,526	ADN53657	*vwb*, *scn*
SaPI68111	16,422	JN689383	*tst*, *sec3*, *sel*
SaPI	15,854	MN450305	*sel*, *sec*
SaPI	15,429	AB716349	*seb*
SaPI	13,477	AB690437	*seb*
SaPI	30,896	MN450303	*seo*, *sem*, *sei*, *sen*, *seg*, *lukE*, *lukD*, *splA*, *splB*, *splC*, *splD*, *splF*
ACME (region Arc)	6174	KC879557	*argR*, *argC*, *argD*, *arcB*, *arcC*
Phi Sa3 (OC8)	42,984	LC129040	*scn*, *sak*, *sea*
Phi Sa3 (252)	43,946	BX571856	*chp*, *sak*, *sea*
Phi SLT in USA300/st. NRS384	46,553	CP027476	*lukS-PV*, *lukF-PV*
Phi Sa2-NARSA676	41,838	OP493558	*lukS-PV*, *lukF-PV*
Phi Sa2-NARSA483	42,927	OP493557	*lukS-PV*, *lukF-PV*
Phi Saeq1 *	42,098	LT671578	*eqscn*, *lukQ*, *lukP*

Abbreviations: SCCmec, staphylococcal chromosomal cassette mec; SaPI, *S. aureus* pathogenicity island; phi, prophage; p, plasmid; * isolated from animal biotypes *S. aureus.*

## Data Availability

No new data were created or analyzed in this study. Data sharing is not applicable to this article.

## Abbreviations

The following abbreviations are used in this manuscript:

aa	number of amino acids
Aaa	two-domain protein autolysin/adhesin
ACME	arginine catabolic mobile element
AD	atopic dermatitis
Agr	global Agr regulatory system
AIP	autoinducing peptide
ArlRS	autolysis-related locus
Atl	major autolysin and adhesin),
Bap	biofilm-associated protein
bp	base pair
CA-MRSA	community acquired MRSA
CA-Sa	community-acquired *S. aureus*
CC	clonal complex
Chp (CHIPS)	chemotaxis-inhibiting protein
Chr	chromosome
ClfA	clumping factor
Cna	collagen binding adhesin
CP	capsular polysaccharide
Eap	extracellular adherence protein
Ebh	extracellular matrix-binding protein
EbpS	elastin binding protein S
Ecb	extracellular complement binding protein)
eDNA	extracellular DNA
Efb	extracellular fibrinogen binding protein
egc	enterotoxin gene cluster
Emp	extracellular matrix protein-binding adhesin
ET	epidermolytic toxin (exfoliatin)
Fnbp	fibronectin binding protein
HA-MRSA	hospital acquired or healthcare acquired MRSA
HA-Sa	hospital-acquired or healthcare-associated *S.aureus*
Hla	alpha toxin (alpha hemolysin)
Hlb	beta toxin (beta hemolysin)
Hld	delta toxin (delta henolysin)
Hlg	gamma toxin (gamma hemolysin)
IL	interleukin
iNOS	nitric oxide synthase isoform
IsdA	iron-regulated surface determinant protein A
LA-MRSA	livestock associated MRSA
LA-Sa	livestock associated *S.aureus*
LTA	lipoteichoic acid
Luk	leukocidin
MIC	minimal inhibitory concentration
MBC	minimal bactericidal concentration
MHC	major histocompatibility complex
MLST	multilocus sequence typing
mRNA	messenger RNA
MRSA	methicillin-resistant *S. aureus*
MSCRAMM	microbial surface components recognizing adhesive matrix molecules
MSSA	methicillin-sensitive *S. aureus*
NAE	North American lineage
p	plasmid
PFGE	pulsed-field gel electrophoresis
PGN	peptidoglycan
phi (Φ)	prophage
PIA	polysaccharide intercellular antigen (PNAG, poly-N-acetylglucosamine)
PNAG	poly-N-acetylglucosamine
PSM	phenol soluble modulin
PVL	Panton-Valentine leukocidin
QQ	quorum quenching
QS	quorum sensing
QSI	quorum sensing inhibitor
Rbf	protein regulator of biofilm formation
RBS	ribosome binding site
RNAII	transcript of the Agr region from promoter P2, containing the genes *agrBDCA*
RNAIII	transcript of the Agr region from promoter P3, contains the *hla* gene
SAE	South American line
SaeRS	*S. aureus* exoprotein expression
SAg	superantigen
SaPI	*S.aureus* pathogenic island
Sar	staphylococcal accessory regulator
Sas	*S. aureus* surface protein
Sbi	*Staphylococcus aureus* binder of IgG
SCCmec	staphylococcal chromosomal cassette mec
Scn (SCIN)	staphylococcal complement inhibitor
Sdr	serine-aspartate repeat-containing protein
SE	staphylococcal enterotoxin
SEl	staphylococcal like enterotoxin
SigB	sigma B
Spa (SpA)	*Staphylococcus aureus* immunoglobulin G binding protein A
SpeG	spermidine N-acetyltransferase
sRNA	small RNA
SSSS	staphylococcal scalded skin syndrome
ST	sequence type
st.	strain
TCR	T-cell receptor
TCS	two-component systems
TSST-1	Toxic Shock Syndrome Toxin 1
VNTR	variable-number tandem repeat
vSa (vSA, GI)	genomic islands
Vwb	von Willebrand factor-binding protein

## References

[1] Morgan Bustamante B.L., Fejerman L., May L., Martínez-López B. (2024). Community-acquired *Staphylococcus aureus* skin and soft tissue infection risk assessment using hotspot analysis and risk maps: The case of California emergency departments. BMC Public Health.

[2] Khan S.A., Gudeta D.D., Chon J., Snippes Vagnone P., Nawaz M.S., Foley S.L., Sung K. (2022). Whole-genome sequences of nine hospital-associated methicillin-susceptible *Staphylococcus aureus* strains. Microbiol. Resour. Announc..

[3] Holtfreter S., Grumann D., Balau V., Barwich A., Kolata J., Goehler A., Weiss S., Holtfreter B., Bauerfeind S.S., Döring P. (2016). Molecular epidemiology of *Staphylococcus aureus* in the general population in northeast Germany: Results of the study of health in Pomerania (SHIP-TREND-0). J. Clin. Microbiol..

[4] Roberts J.C., Cannons A.C., Amuso P.T., Cattani J. (2006). Virtual digest identification of secondary enzymes for use in pulsed-field gel electrophoresis of *Staphylococcus aureus*. J. Microbiol. Methods.

[5] Lakhundi S., Zhang K. (2018). Methicillin-resistant *Staphylococcus aureus*: Molecular characterization, evolution, and epidemiology. Clin. Microbiol. Rev..

[6] Mlynarczyk-Bonikowska B., Kowalewski C., Krolak-Ulinska A., Marusza W. (2022). Molecular mechanisms of drug resistance in *Staphylococcus aureus*. Int. J. Mol. Sci..

[7] Novick R.P., Fischetti V.A., Novick R.P., Farretti J.J., Partnoy D.A., Rood J.I. (2006). Staphylococcal pathogenesis and pathogenicity factors: Genetic and regulation. Gram-Positive Pathogens.

[8] Gupta R.K., Luong T.T., Lee C.Y. (2015). RNAIII of the *Staphylococcus aureus* agr system activates global regulator MgrA by stabilizing mRNA. Proc. Natl. Acad. Sci. USA.

[9] Sun L., Wu D., Chen Y., Wang Q., Wang H., Yu Y. (2017). Characterization of a PVL-negative community-acquired methicillin-resistant *Staphylococcus aureus* strain of sequence type 88 in China. Int. J. Med. Microbiol..

[10] Uehara Y., Sasaki T., Baba T., Lu Y., Imajo E., Sato Y., Tanno S., Furuichi M., Kawada M., Hiramatsu K. (2019). Regional outbreak of community-associated methicillin-resistant *Staphylococcus aureus* ST834 in Japanese children. BMC Infect. Dis..

[11] Jiang J.-H., Cameron D.R., Nethercott C., Aires-de-Sousa M., Peleg A.Y. (2023). Virulence attributes of successful methicillin-resistant *Staphylococcus aureus* lineages. Clin. Microbiol. Rev..

[12] Chua K.Y., Monk I.R., Lin Y.H., Seemann T., Tuck K.L., Porter J.L., Stepnell J., Coombs G.W., Davies J.K., Stinear T.P. (2014). Hyperexpression of alpha-hemolysin explains enhanced virulence of sequence type 93 community-associated methicillin-resistant *Staphylococcus aureus*. BMC Microbiol..

[13] Montgomery C.P., Boyle-Vavra S., Daum R.S. (2010). Importance of the global regulators agr and saeRS in the pathogenesis of CA-MRSA USA300 infection. PLoS ONE.

[14] Rolo J., Miragaia M., Turlej-Rogacka A., Empel J., Bouchami O., Faria N.A., Tavares A., Hryniewicz W., Fluit A.C., de Lencastre H. (2012). High genetic diversity among community-associated *Staphylococcus aureus* in Europe: Results from a multicenter study. PLoS ONE.

[15] Szymanek-Majchrzak K., Młynarczyk G. (2022). Genomic insights of first ermB-positive ST338-SCCmecV_T_/CC59 Taiwan clone of community-associated methicillin-resistant *Staphylococcus aureus* in Poland. Int. J. Mol. Sci..

[16] Yu L., Hisatsune J., Hirakawa H., Mizumachi E., Toyoda A., Yahara K., Sugai M. (2017). Complete genome sequence of super biofilm-elaborating *Staphylococcus aureus* isolated in Japan. Genome Announc..

[17] Yonemoto K., Chiba A., Sugimoto S., Sato C., Saito M., Kinjo Y., Marumo K., Mizunoe Y. (2019). Redundant and distinct roles of secreted protein Eap and cell wall-anchored protein SasG in biofilm formation and pathogenicity of *Staphylococcus aureus*. Infect. Immun..

[18] De Backer S., Xavier B.B., Vanjari L., Coppens J., Lammens C., Vemu L., Carevic B., Hryniewicz W., Jorens P., Kumar-Singh S. (2019). Remarkable geographical variations between India and Europe in carriage of the staphylococcal surface protein-encoding sasX/sesI and in the population structure of methicillin-resistant *Staphylococcus aureus* belonging to clonal complex 8. Clin. Microbiol. Infect..

[19] Savolainen K., Paulin L., Westerlund-Wikström B., Foster T.J., Korhonen T.K., Kuusela P. (2001). Expression of *pls*, a gene closely associated with the *mecA* gene of methicillin-resistant *Staphylococcus aureus*, prevents bacterial adhesion in vitro. Infect. Immun..

[20] Thurlow L.R., Joshi G.S., Clark J.R., Spontak J.S., Neely C.J., Maile R., Richardson A.R. (2013). Functional modularity of the arginine catabolic mobile element contributes to the success of USA300 methicillin-resistant *Staphylococcus aureus*. Cell Host Microbe.

[21] Joshi G.S., Spontak J.S., Klapper D.G., Richardson A.R. (2011). Arginine catabolic mobile element encoded *speG* abrogates the unique hypersensitivity of *Staphylococcus aureus* to exogenous polyamines. Mol. Microbiol..

[22] Sabat A.J., Köck R., Akkerboom V., Hendrix R., Skov R.L., Becker K., Friedrich A.W. (2013). Novel organization of the arginine catabolic mobile element and staphylococcal cassette chromosome mec composite island and its horizontal transfer between distinct *Staphylococcus aureus* genotypes. Antimicrob. Agents Chemother..

[23] Diep B.A., Gill S.R., Chang R.F., Phan T.H., Chen J.H., Davidson M.G., Lin F., Lin J., Carleton H.A., Mongodin E.F. (2006). Complete genome sequence of USA300, an epidemic clone of community-acquired meticillin-resistant *Staphylococcus aureus*. Lancet.

[24] Kawaguchiya M., Urushibara N., Ghosh S., Kuwahara O., Morimoto S., Ito M., Kudo K., Kobayashi N. (2013). Genetic diversity of emerging Panton-Valentine leukocidine/arginine catabolic mobile element (ACME)-positive ST8 SCCmec-IVa meticillin-resistant *Staphylococcus aureus* (MRSA) strains and ACME-positive CC5 (ST5/ST764) MRSA strains in Northern Japan. J. Med. Microbiol..

[25] Aung M.S., Kawaguchiya M., Urushibara N., Sumi A., Ito M., Kudo K., Morimoto S., Hosoya S., Kobayashi N. (2017). Molecular characterization of methicillin-resistant *Staphylococcus aureus* from outpatients in northern Japan: Increasing tendency of ST5/ST764 MRSA-IIa with arginine catabolic mobile element. Microb. Drug Resist..

[26] Urushibara N., Aung M.S., Kawaguchiya M., Kobayashi N. (2020). Novel staphylococcal cassette chromosome mec (SCCmec) type XIV (5A) and a truncated SCCmec element in SCC composite islands carrying *speG* in ST5 MRSA in Japan. J. Antimicrob. Chemother..

[27] Morrisette T., Stamper K.C., Lev K.L., Kebriaei R., Holger D.J., Abdul-Mutakabbir J.C., Kunz Coyne A.J., Rybak M.J. (2023). Evaluation of omadacycline alone and in combination with rifampin against *Staphylococcus aureus* and *Staphylococcus epidermidis* in an in vitro pharmacokinetic/pharmacodynamic biofilm model. Antimicrob. Agents Chemother..

[28] Ji X., Krüger H., Tao J., Wang Y., Feßler A.T., Bai R., Wang S., Dong Y., Shen J., Wang Y. (2021). Comparative analysis of genomic characteristics, fitness and virulence of MRSA ST398 and ST9 isolated from China and Germany. Emerg. Microbes Infect..

[29] Zhao X., Chlebowicz-Flissikowska M.A., Wang M., Vera Murguia E., de Jong A., Becher D., Maaß S., Buist G., van Dijl J.M. (2020). Exoproteomic profiling uncovers critical determinants for virulence of livestock-associated and human-originated *Staphylococcus aureus* ST398 strains. Virulence.

[30] Amdahl H., Haapasalo K., Tan L., Meri T., Kuusela P.I., van Strijp J.A., Rooijakkers S., Jokiranta T.S. (2017). Staphylococcal protein Ecb impairs complement receptor-1 mediated recognition of opsonized bacteria. PLoS ONE.

[31] Senok A.C., Somily A.M., Slickers P., Raji M.A., Garaween G., Shibl A., Monecke S., Ehricht R. (2017). Investigating a rare methicillin-resistant *Staphylococcus aureus* strain: First description of genome sequencing and molecular characterization of CC15-MRSA. Infect. Drug Resist..

[32] Jongerius I., Köhl J., Pandey M.K., Ruyken M., van Kessel K.P., van Strijp J.A., Rooijakkers S.H. (2007). Staphylococcal complement evasion by various convertase-blocking molecules. J. Exp. Med..

[33] van Wamel W.J., Rooijakkers S.H., Ruyken M., van Kessel K.P., van Strijp J.A. (2006). The innate immune modulators staphylococcal complement inhibitor and chemotaxis inhibitory protein of *Staphylococcus aureus* are located on beta-hemolysin-converting bacteriophages. J. Bacteriol..

[34] McCarthy A.J., van Wamel W., Vandendriessche S., Larsen J., Denis O., Garcia-Graells C., Uhlemann A.C., Lowy F.D., Skov R., Lindsay J.A. (2012). *Staphylococcus aureus* CC398 clade associated with human-to-human transmission. Appl. Environ. Microbiol..

[35] Smith E.J., Corrigan R.M., van der Sluis T., Gründling A., Speziale P., Geoghegan J.A., Foster T.J. (2012). The immune evasion protein Sbi of *Staphylococcus aureus* occurs both extracellularly and anchored to the cell envelope by binding lipoteichoic acid. Mol. Microbiol..

[36] Foster T.J., Geoghegan J.A., Ganesh V.K., Höök M. (2014). Adhesion, invasion and evasion: The many functions of the surface proteins of *Staphylococcus aureus*. Nat. Rev. Microbiol..

[37] Holden M.T., Feil E.J., Lindsay J.A., Peacock S.J., Day N.P., Enright M.C., Foster T.J., Moore C.E., Hurst L., Atkin R. (2004). Complete genomes of two clinical *Staphylococcus aureus* strains: Evidence for the rapid evolution of virulence and drug resistance. Proc. Natl. Acad. Sci. USA.

[38] Chaffin D.O., Taylor D., Skerrett S.J., Rubens C.E. (2012). Changes in the *Staphylococcus aureus* transcriptome during early adaptation to the lung. PLoS ONE.

[39] Zhang L., Jacobsson K., Vasi J., Lindberg M., Frykberg L. (1998). A second IgG-binding protein in *Staphylococcus aureus*. Microbiology.

[40] Wan T.W., Khokhlova O.E., Iwao Y., Higuchi W., Hung W.C., Reva I.V., Singur O.A., Gostev V.V., Sidorenko S.V., Peryanova O.V. (2016). Complete circular genome sequence of successful ST8/SCCmecIV community-associated methicillin-resistant *Staphylococcus aureus* (OC8) in Russia: One-megabase genomic inversion, IS256’s spread, and evolution of Russia ST8-IV. PLoS ONE.

[41] Viana D., Blanco J., Tormo-Más M.A., Selva L., Guinane C.M., Baselga R., Corpa J., Lasa I., Novick R.P., Fitzgerald J.R. (2010). Adaptation of *Staphylococcus aureus* to ruminant and equine hosts involves SaPI-carried variants of von Willebrand factor-binding protein. Mol. Microbiol..

[42] Koop G., Vrieling M., Storisteanu D.M., Lok L.S., Monie T., van Wigcheren G., Raisen C., Ba X., Gleadall N., Hadjirin N. (2017). Identification of LukPQ, a novel, equid-adapted leukocidin of *Staphylococcus aureus*. Sci. Rep..

[43] White J., Herman A., Pullen A.M., Kubo R., Kappler J.W., Marrack P. (1989). The Vbeta-specific superantigen staphylococcal enterotoxin B: Stimulation of mature T cells and clonal deletion in neonatal mice. Cell.

[44] Brouillard J.N., Günther S., Varma A.K., Gryski I., Herfst C.A., Rahman A.K., Leung D.Y., Schlievert P.M., Madrenas J., Sundberg E.J. (2007). Crystal structure of the streptococcal superantigen SpeI and functional role of a novel loop domain in T cell activation by group V superantigens. J. Mol. Biol..

[45] Marrack P., Kappler J. (1990). The Staphylococcal enterotoxins and their relatives. Science.

[46] Levinson W. (2020). Review of Medical Microbiology and Immunology.

[47] Li H., Llera A., Malchiodi E.L., Mariuzza R.A. (1999). The structural basis of T cell activation by superantigens. Annu. Rev. Immunol..

[48] Girma A. (2024). *Staphylococcus aureus*: Current perspectives on molecular pathogenesis and virulence. Cell Surf..

[49] Seo K.S. (2016). Superantigens. Methods and Protocols.

[50] Huang J., Xu Y. (2023). Autoimmunity: A new focus on nasal polyps. Int. J. Mol. Sci..

[51] Tuffs S.W., Haeryfar S.M.M., McCormick J.K. (2018). Manipulation of innate and adaptive immunity by Staphylococcal superantigens. Pathogens.

[52] Spaulding A.R., Salgado-Pabón W., Kohler P.L., Horswill A.R., Leung D.Y.M., Schlievert P.M. (2013). Staphylococcal and streptococcal superantigen exotoxins. Clin. Microbiol. Rev..

[53] Spoor L.E., Richardson E., Richards A.C., Wilson G.J., Mendonca C., Gupta R.K., McAdam P.R., Nutbeam-Tuffs S., Black N.S., O’GAra J.P. (2015). Recombination-mediated remodelling of host–pathogen interactions during *Staphylococcus aureus* niche adaptation. Microb. Genom..

[54] Fraser J.D., Proft T. (2008). The bacterial superantigen and superantigen-like proteins. Immunol. Rev..

[55] Seo K.S., Park J.Y., Terman D.S., Bohach G.A. (2010). A quantitative real time PCR method to analyze T cell receptor Vbeta subgroup expansion by staphylococcal superantigens. J. Transl. Med..

[56] Noli Truant S., Redolfi D.M., Sarratea M.B., Malchiodi E.L., Fernández M.M. (2022). Superantigens, a paradox of the immune response. Toxins.

[57] Fleischer B., Gerlach D., Fuhrmann A., Schmidt K.H. (1995). Superantigens and pseudosuperantigens of gram-positive cocci. Med. Microbiol. Immunol..

[58] Thomas D., Dauwalder O., Brun V., Badiou C., Ferry T., Etienne J., Vandenesch F., Lina G. (2009). *Staphylococcus aureus* superantigens elicit redundant and extensive human Vbeta patterns. Infect. Immun..

[59] Krakauer T. (2019). Staphylococcal superantigens: Pyrogenic toxins induce toxic shock. Toxins.

[60] Choi Y.W., Kotzin B., Herron L., Callahan J., Marrack P., Kappler J. (1989). Interaction of *Staphylococcus aureus* toxin “superantigens” with human T cells. Proc. Natl. Acad. Sci. USA.

[61] Deringer J.R., Ely R.J., Monday S.R., Stauffacher C.V., Bohach G.A. (1997). Vbeta-dependent stimulation of bovine and human T cells by host-specific staphylococcal enterotoxins. Infect. Immun..

[62] Deringer J.R., Ely R.J., Stauffacher C.V., Bohach G.A. (1996). Subtype-specific interactions of type C staphylococcal enterotoxins with the T-cell receptor. Mol. Microbiol..

[63] Petersson K., Pettersson H., Skartved N.J., Walse B., Forsberg G. (2003). Staphylococcal enterotoxin H induces V alpha-specific expansion of T cells. J. Immunol..

[64] Petersson K., Håkansson M., Nilsson H., Forsberg G., Svensson L.A., Liljas A., Walse B. (2001). Crystal structure of a superantigen bound to MHC class II displays zinc and peptide dependence. EMBO J..

[65] Hakansson M., Petersson K., Nilsson H., Forsberg G., Björk P., Antonsson P., Svensson L.A. (2000). The crystal structure of staphylococcal enterotoxin H: Implications for binding properties to MHC class II and TcR molecules. J. Mol. Biol..

[66] Zhu Z., Hu Z., Li S., Fang R., Ono H.K., Hu D.L. (2023). Molecular characteristics and pathogenicity of *Staphylococcus aureus* exotoxins. Int. J. Mol. Sci..

[67] Kim J., Urban R.G., Strominger J.L., Wiley D.C. (1994). Toxic shock syndrome toxin-1 complexed with a class II major histocompatibility molecule HLA DR1. Science.

[68] Jardetzky T.S., Brown J.H., Gorga J.C., Stern L.J., Urban R.G., Chi Y.I., Stauffacher C., Strominger J.L., Wiley D.C. (1994). Three-dimensional structure of a human class II histocompatibility molecule complexed with superantigen. Nature.

[69] Sundberg E.J., Li H., Llera A.S., McCormick J.K., Tormo J., Schlievert P.M., Karjalainen K., Mariuzza R.A. (2002). Structures of two streptococcal superantigens bound to TCR beta chains reveal diversity in the architecture of T cell signaling complexes. Structure.

[70] Ma R., Qiu S., Jiang Q., Sun H., Xue T., Cai G., Sun B. (2017). AI-2 quorum sensing negatively regulates rbf expression and biofilm formation in *Staphylococcus aureus*. Int. J. Med. Microbiol..

[71] Mlynarczyk A., Mlynarczyk B., Kmera-Muszynska M., Majewski S., Mlynarczyk G. (2009). Mechanisms of the resistance and tolerance to beta-lactam and glycopeptide antibiotics in pathogenic gram-positive cocci. Mini Rev. Med. Chem..

[72] Mlynarczyk B., Mlynarczyk A., Kmera-Muszynska M., Majewski S., Mlynarczyk G. (2010). Mechanisms of resistance to antimicrobial drugs in pathogenic Gram-positive cocci. Mini Rev. Med. Chem..

[73] Thakker M., Park J.S., Carey V., Lee J.C. (1998). *Staphylococcus aureus* serotype 5 capsular polysaccharide is antiphagocytic and enhances bacterial virulence in a murine bacteremia model. Infect. Immun..

[74] Nilsson I.M., Lee J.C., Bremell T., Rydén C., Tarkowski A. (1997). The role of staphylococcal polysaccharide microcapsule expression in septicemia and septic arthritis. Infect. Immun..

[75] Portolés M., Kiser K.B., Bhasin N., Chan K.H.N., Lee J.C. (2001). *Staphylococcus aureus* cap5o has udp-mannac dehydrogenase activity and is essential for capsule expression. Infect. Immun..

[76] Lehmann E., van Dalen R., Gritsch L., Slavetinsky C., Korn N., Rohmer C., Krause D., Peschel A., Weidenmaier C., Wolz C. (2024). The capsular polysaccharide obstructs wall teichoic acid functions in *Staphylococcus aureus*. J. Infect. Dis..

[77] David M.Z., Daum R.S. (2010). Community-associated methicillin-resistant *Staphylococcus aureus*: Epidemiology and clinical consequences of an emerging epidemic. Clin. Microbiol. Rev..

[78] Nanra J.S., Buitrago S.M., Crawford S., Ng J., Fink P.S., Hawkins J., Scully I.L., McNeil L.K., Aste-Amézaga J.M., Cooper D. (2013). Capsular polysaccharides are an important immune evasion mechanism for *Staphylococcus aureus*. Hum. Vaccines Immunother..

[79] Osterlid K.E., Cergano R., Overkleeft H.S., van der Marel G.A., Codée J.D.C. (2025). Synthesis of a Set of *Staphylococcus aureus* capsular polysaccharide type 1 oligosaccharides carrying taurine esters. Chemistry.

[80] Visansirikul S., Kolodziej S.A., Demchenko A.V. (2020). *Staphylococcus aureus* capsular polysaccharides: A structural and synthetic perspective. Org. Biomol. Chem..

[81] Rausch M., Deisinger J.P., Ulm H., Müller A., Li W., Hardt P., Wang X., Li X., Sylvester M., Engeser M. (2019). Coordination of capsule assembly and cell wall biosynthesis in *Staphylococcus aureus*. Nat. Commun..

[82] Keinhörster D., George S.E., Weidenmaier C., Wolz C. (2019). Function and regulation of *Staphylococcus aureus* wall teichoic acids and capsular polysaccharides. Int. J. Med. Microbiol..

[83] Keinhörster D., Salzer A., Duque-Jaramillo A., George S.E., Marincola G., Lee J.C., Weidenmaier C., Wolz C. (2019). Revisiting the regulation of the capsular polysaccharide biosynthesis gene cluster in *Staphylococcus aureus*. Mol. Microbiol..

[84] Guo Z., Peng H., Shang W., Yang Y., Hu Z., Rao Y., Huang X., Dou J., Xu Z., Rao X. (2025). *WalK* (*S*221P) mutation promotes the production of *Staphylococcus aureus* capsules through an MgrA-dependent pathway. Microorganisms.

[85] Osterlid K.E., Sorieul C., Unione L., Li S., García-Sepúlveda C., Carboni F., Del Bino L., Berni F., Arda A., Overkleeft H.S. (2025). Long, synthetic *Staphylococcus aureus* type 8 capsular oligosaccharides reveal structural epitopes for effective immune recognition. J. Am. Chem. Soc..

[86] Gupta R.K., Alba J., Xiong Y.Q., Bayer A.S., Lee C.Y. (2013). MgrA activates expression of capsule genes, but not the alpha-toxin gene in experimental *Staphylococcus aureus* endocarditis. J. Infect. Dis..

[87] Boyle-Vavra S., Li X., Alam M.T., Read T.D., Sieth J., Cywes-Bentley C., Dobbins G., David M.Z., Kumar N., Eells S.J. (2015). USA300 and USA500 clonal lineages of *Staphylococcus aureus* do not produce a capsular polysaccharide due to conserved mutations in the cap5 locus. mBio.

[88] Wu X., Wang H., Xiong J., Yang G.X., Hu J.F., Zhu Q., Chen Z. (2024). *Staphylococcus aureus* biofilm: Formulation, regulatory, and emerging natural products-derived therapeutics. Biofilm.

[89] Sedarat Z., Taylor-Robinson A.W. (2022). Biofilm formation by pathogenic bacteria: Applying a *Staphylococcus aureus* model to appraise potential targets for therapeutic intervention. Pathogens.

[90] Peng Q., Tang X., Dong W., Sun N., Yuan W. (2022). A review of biofilm formation of *Staphylococcus aureus* and its regulation mechanism. Antibiotics.

[91] Sabat A., Melles D.C., Martirosian G., Grundmann H., van Belkum A., Hryniewicz W. (2006). Distribution of the serine-aspartate repeat protein-encoding *sdr* genes among nasal-carriage and invasive *Staphylococcus aureus* strains. J. Clin. Microbiol..

[92] Sitkiewicz I., Babiak I., Hryniewicz W. (2011). Characterization of transcription within *sdr* region of *Staphylococcus aureus*. Antonie Van Leeuwenhoek.

[93] Cucarella C., Solano C., Valle J., Amorena B., Lasa I., Penadés J.R. (2001). Bap, a *Staphylococcus aureus* surface protein involved in biofilm formation. J. Bacteriol..

[94] Ubeda C., Tormo M.A., Cucarella C., Trotonda P., Foster T.J., Lasa I., Penadés J.R. (2003). Sip, an integrase protein with excision, circularization and integration activities, defines a new family of mobile *Staphylococcus aureus* pathogenicity islands. Mol. Microbiol..

[95] Togashi A., Aung M.S., Yoto Y., Tsugawa T., Sueoka H., Kawaguchiya M., Tsutsumi H., Kobayashi N. (2017). Septic arthritis caused by an emerging ST121 methicillin-susceptible, PVL-negative *Staphylococcus aureus* harbouring a variant of bone sialoprotein-binding protein gene. New Microbes New Infect..

[96] Kuhn G., Francioli P., Blanc D.S. (2006). Evidence for clonal evolution among highly polymorphic genes in methicillin-resistant *Staphylococcus aureus*. J. Bacteriol..

[97] Cheng C., Jiang T., Zhang D., Wang H., Fang T., Li C. (2023). Attachment characteristics and kinetics of biofilm formation by *Staphylococcus aureus* on ready-to-eat cooked beef contact surfaces. J. Food Sci..

[98] Konduri R., Saiabhilash C.R., Shivaji S. (2021). Biofilm-forming potential of ocular fluid *Staphylococcus aureus* and *Staphylococcus epidermidis* on ex vivo human corneas from attachment to dispersal phase. Microorganisms.

[99] Guo H., Tong Y., Cheng J., Abbas Z., Li Z., Wang J., Zhou Y., Si D., Zhang R. (2022). Biofilm and small colony variants-an update on *Staphylococcus aureus* strategies toward drug resistance. Int. J. Mol. Sci..

[100] Flemming H.C., Wingender J., Szewzyk U., Steinberg P., Rice S.A., Kjelleberg S. (2016). Biofilms: An emergent form of bacterial life. Nat. Rev. Microbiol..

[101] Ciofu O., Moser C., Jensen P.Ø., Høiby N. (2022). Tolerance and resistance of microbial biofilms. Nat. Rev. Microbiol..

[102] Chopra S., Harjai K., Chhibber S. (2015). Antibiotic susceptibility of ica-positive and ica-negative MRSA in different phases of biofilm growth. J. Antibiot..

[103] Xu L., Zhang X., Wang W., Shen J., Ma K., Wang H., Xue T. (2025). The global regulator SpoVG is involved in biofilm formation and stress response in foodborne *Staphylococcus aureus*. Int. J. Food Microbiol..

[104] Hernandez-Cuellar E., Tsuchiya K., Valle-Ríos R., Medina-Contreras O. (2023). Differences in biofilm formation by methicillin-resistant and methicillin-susceptible *Staphylococcus aureus* strains. Diseases.

[105] Cue D., Lei M.G., Luong T.T., Kuechenmeister L., Dunman P.M., O’Donnell S., Rowe S., O’Gara J.P., Lee C.Y. (2009). Rbf promotes biofilm formation by *Staphylococcus aureus* via repression of icaR, a negative regulator of icaADBC. J. Bacteriol..

[106] Nguyen H.T.T., Nguyen T.H., Otto M. (2020). The staphylococcal exopolysaccharide PIA—Biosynthesis and role in biofilm formation, colonization, and infection. Comput. Struct. Biotechnol. J..

[107] Schaeffer C.R., Woods K.M., Longo G.M., Kiedrowski M.R., Paharik A.E., Büttner H., Christner M., Boissy R.J., Horswill A.R., Rohde H. (2015). Accumulation-associated protein enhances *Staphylococcus epidermidis* biofilm formation under dynamic conditions and is required for infection in a rat catheter model. Infect. Immun..

[108] Lei M.G., Cue D., Roux C.M., Dunman P.M., Lee C.Y. (2011). Rsp inhibits attachment and biofilm formation by repressing fnbA in *Staphylococcus aureus* MW2. J. Bacteriol..

[109] Porayath C., Suresh M.K., Biswas R., Nair B.G., Mishra N., Pal S. (2018). Autolysin mediated adherence of *Staphylococcus aureus* with fibronectin, gelatin and heparin. Int. J. Biol. Macromol..

[110] Hirschhausen N., Schlesier T., Peters G., Heilmann C. (2012). Characterization of the modular design of the autolysin/adhesin Aaa from *Staphylococcus aureus*. PLoS ONE.

[111] Foster T.J. (2019). Surface proteins of *Staphylococcus aureus*. Microbiol. Spectr..

[112] Foster T.J. (2019). The MSCRAMM family of cell-wall-anchored surface proteins of gram-positive cocci. Trends Microbiol..

[113] Viela F., Speziale P., Pietrocola G., Dufrêne Y.F. (2019). Bacterial pathogens under high-tension: *Staphylococcus aureus* adhesion to von Willebrand factor is activated by force. Microb. Cell.

[114] Lamret F., Varin-Simon J., Velard F., Terryn C., Mongaret C., Colin M., Gangloff S.C., Reffuveille F. (2021). *Staphylococcus aureus* strain-dependent biofilm formation in bone-like environment. Front. Microbiol..

[115] Tam K., Torres V.J. (2019). *Staphylococcus aureus* secreted toxins and extracellular enzymes. Microbiol. Spectr..

[116] Wilke G.A., Bubeck Wardenburg J. (2010). Role of a disintegrin and metalloprotease 10 in *Staphylococcus aureus* alpha-hemolysin-mediated cellular injury. Proc. Natl. Acad. Sci. USA.

[117] Popov L.M., Marceau C.D., Starkl P.M., Lumb J.H., Shah J., Guerrera D., Cooper R.L., Merakou C., Bouley D.M., Meng W. (2015). The adherens junctions control susceptibility to *Staphylococcus aureus* alpha-toxin. Proc. Natl. Acad. Sci. USA.

[118] Winter S.V., Zychlinsky A., Bardoel B.W. (2016). Genome-wide CRISPR screen reveals novel host factors required for *Staphylococcus aureus* alpha-hemolysin-mediated toxicity. Sci. Rep..

[119] von Hoven G., Rivas A.J., Neukirch C., Klein S., Hamm C., Qin Q., Meyenburg M., Füser S., Saftig P., Hellmann N. (2016). Dissecting the role of ADAM10 as a mediator of *Staphylococcus aureus* alpha-toxin action. Biochem. J..

[120] Lemjabbar H., Basbaum C. (2002). Platelet-activating factor receptor and ADAM10 mediate responses to *Staphylococcus aureus* in epithelial cells. Nat. Med..

[121] Schulz B., Pruessmeyer J., Maretzky T., Ludwig A., Blobel C.P., Saftig P., Reiss K. (2008). ADAM10 regulates endothelial permeability and T-cell transmigration by proteolysis of vascular endothelial cadherin. Circ. Res..

[122] Maretzky T., Reiss K., Ludwig A., Buchholz J., Scholz F., Proksch E., de Strooper B., Hartmann D., Saftig P. (2005). ADAM10 mediates E-cadherin shedding and regulates epithelial cell-cell adhesion, migration, and beta-catenin translocation. Proc. Natl. Acad. Sci. USA.

[123] Colciaghi F., Borroni B., Pastorino L., Marcello E., Zimmermann M., Cattabeni F., Padovani A., Di Luca M. (2002). [alpha]-secretase ADAM10 as well as [alpha] APPs is reduced in platelets and CSF of Alzheimer disease patients. Mol. Med..

[124] Oliveira D., Borges A., Simões M. (2018). *Staphylococcus aureus* toxins and their molecular activity in infectious diseases. Toxins.

[125] Kong E.F., Tsui C., Kucharíková S., Andes D., Van Dijck P., Jabra-Rizk M.A. (2016). Commensal protection of *Staphylococcus aureus* against antimicrobials by *Candida albicans* biofilm matrix. mBio.

[126] Li C., Wang A., Wu Y., Gulbins E., Grassmé H., Zhao Z. (2019). Acid sphingomyelinase-ceramide system in bacterial infections. Cell Physiol. Biochem..

[127] Becker K.A., Fahsel B., Kemper H., Mayeres J., Li C., Wilker B., Keitsch S., Soddemann M., Sehl C., Kohnen M. (2017). *Staphylococcus aureus* alpha-toxin disrupts endothelial-cell tight junctions via acid sphingomyelinase and ceramide. Infect. Immun..

[128] Ma J., Gulbins E., Edwards M.J., Caldwell C.C., Fraunholz M., Becker K.A. (2017). *Staphylococcus aureus* α-toxin induces inflammatory cytokines via lysosomal acid sphingomyelinase and ceramides. Cell Physiol. Biochem..

[129] Di Bella S., Marini B., Stroffolini G., Geremia N., Giacobbe D.R., Campanile F., Bartoletti M., Alloisio G., Di Risio L., Viglietti G. (2025). The virulence toolkit of *Staphylococcus aureus*: A comprehensive review of toxin diversity, molecular mechanisms, and clinical implications. Eur. J. Clin. Microbiol. Infect. Dis..

[130] Goerke C., Pantucek R., Holtfreter S., Schulte B., Zink M., Grumann D., Bröker B.M., Doskar J., Wolz C. (2009). Diversity of prophages in dominant *Staphylococcus aureus* clonal lineages. J. Bacteriol..

[131] Tajima A., Iwase T., Shinji H., Seki K., Mizunoe Y. (2009). Inhibition of endothelial interleukin-8 production and neutrophil transmigration by *Staphylococcus aureus* beta-hemolysin. Infect. Immun..

[132] Yoong P., Torres V.J. (2013). The effects of *Staphylococcus aureus* leukotoxins on the host: Cell lysis and beyond. Curr. Opin. Microbiol..

[133] Vrieling M., Boerhout E.M., van Wigcheren G.F., Koymans K.J., Mols-Vorstermans T.G., de Haas C.J.C., Aerts P.C., Daemen I.J.J.M., van Kessel K.P.M., Koets A.P. (2016). LukMF’ is the major secreted leukocidin of bovine *Staphylococcus aureus* and is produced in vivo during bovine mastitis. Sci. Rep..

[134] Yamada T., Tochimaru N., Nakasuji S., Hata E., Kobayashi H., Eguchi M., Kaneko J., Kamio Y., Kaidoh T., Takeuchi S. (2005). Leukotoxin family genes in *Staphylococcus aureus* isolated from domestic animals and prevalence of *lukM-lukF-PV* genes by bacteriophages in bovine isolates. Vet. Microbiol..

[135] Boakes E., Kearns A.M., Ganner M., Perry C., Hill R.L., Ellington M.J. (2011). Distinct bacteriophages encoding Panton-Valentine leukocidin (PVL) among international methicillin-resistant *Staphylococcus aureus* clones harboring PVL. J. Clin. Microbiol..

[136] Kuroda M., Ohta T., Uchiyama I., Baba T., Yuzawa H., Kobayashi I., Cui L., Oguchi A., Aoki K., Nagai Y. (2001). Whole genome sequencing of meticillin-resistant *Staphylococcus aureus*. Lancet.

[137] Spaan A.N., Reyes-Robles T., Badiou C., Cochet S., Boguslawski K.M., Yoong P., Day C.J., de Haas C.J.C., van Kessel K.P.M., Vandenesch F. (2015). *Staphylococcus aureus* targets the Duffy antigen receptor for chemokines (DARC) to lyse erythrocytes. Cell Host Microbe.

[138] Noda M., Hirayama T., Kato I., Matsuda F. (1980). Crystallization and properties of staphylococcal leukocidin. Biochim. Biophys. Acta.

[139] Alonzo F., Torres V.J. (2014). The bicomponent pore-forming leucocidins of *Staphylococcus aureus*. Microbiol. Mol. Biol. Rev..

[140] Yamashita K., Kawai Y., Tanaka Y., Hirano N., Kaneko J., Tomita N., Ohta M., Kamio Y., Yao M., Tanaka I. (2011). Crystal structure of the octameric pore of staphylococcal gamma-hemolysin reveals the beta-barrel pore formation mechanism by two components. Proc. Natl. Acad. Sci. USA.

[141] Yamashita D., Sugawara T., Takeshita M., Kaneko J., Kamio Y., Tanaka I., Tanaka Y., Yao M. (2014). Molecular basis of transmembrane beta-barrel formation of staphylococcal pore-forming toxins. Nat. Commun..

[142] Spaan A.N., van Strijp J.A.G., Torres V.J. (2017). Leukocidins: Staphylococcal bi-component pore-forming toxins find their receptors. Nat. Rev. Microbiol..

[143] Mehlin C., Headley C.M., Klebanoff S.J. (1999). An inflammatory polypeptide complex from *Staphylococcus epidermidis*: Isolation and characterization. J. Exp. Med..

[144] Wang R., Braughton K.R., Kretschmer D., Bach T.H.L., Queck S.Y., Li M., Kennedy A.D., Dorward D.W., Klebanoff S.J., Peschel A. (2007). Identification of novel cytolytic peptides as key virulence determinants for community-associated MRSA. Nat. Med..

[145] Trouillet-Assant S., Lelièvre L., Martins-Simões P., Gonzaga L., Tasse J., Valour F., Rasigade J.P., Vandenesch F., Muniz Guedes R.L., Ribeiro de Vasconcelos A.T. (2016). Adaptive processes of *Staphylococcus aureus* isolates during the progression from acute to chronic bone and joint infections in patients. Cell Microbiol..

[146] Kaito C., Saito Y., Ikuo M., Omae Y., Mao H., Nagano G., Fujiyuki T., Numata S., Han X., Obata K. (2013). Mobile genetic element SCCmec-encoded *psm-mec* RNA suppresses translation of *agrA* and attenuates MRSA virulence. PLoS Pathog..

[147] Peschel A., Otto M. (2013). Phenol-soluble modulins and staphylococcal infection. Nat. Rev. Microbiol..

[148] Kretschmer D., Gleske A.K., Rautenberg M., Wang R., Köberle M., Bohn E., Schöneberg T., Rabiet M.J., Boulay F., Klebanoff S.J. (2010). Human formyl peptide receptor 2 senses highly pathogenic *Staphylococcus aureus*. Cell Host Microbe.

[149] Periasamy S., Joo H.S., Duong A.C., Bach T.M.L., Tan V.Y., Chatterjee S.S., Cheung G.Y.C., Otto M. (2012). How *Staphylococcus aureus* biofilms develop their characteristic structure. Proc. Natl. Acad. Sci. USA.

[150] Tamai M., Yamazaki Y., Ito T., Nakagawa S., Nakamura Y. (2023). Pathogenic role of the staphylococcal accessory gene regulator quorum sensing system in atopic dermatitis. Front. Cell Infect. Microbiol..

[151] Nakamura Y., Oscherwitz J., Cease K.B., Chan S.M., Muñoz-Planillo R., Hasegawa M., Villaruz A.E., Cheung G.Y., McGavin M.J., Travers J.B. (2013). Staphylococcus δ-toxin induces allergic skin disease by activating mast cells. Nature.

[152] Todd J., Fishaut M., Kapral F., Welch T. (1978). Toxic-shock syndrome associated with phage-group-I staphylococci. Lancet.

[153] Majewska A., Mlynarczyk-Bonikowska B. (2022). 40 Years after the registration of acyclovir: Do we need new anti-herpetic drugs?. Int. J. Mol. Sci..

[154] Mlynarczyk-Bonikowska B., Majewska A., Malejczyk M., Mlynarczyk G., Majewski S. (2013). Antiviral medication in sexually transmitted diseases. part I: HSV, HPV. Mini Rev. Med. Chem..

[155] Touaitia R., Ibrahim N.A., Abdullah Almuqri E., Basher N.S., Idres T., Touati A. (2025). Toxic shock syndrome toxin-1 (TSST-1) in *Staphylococcus aureus*: Prevalence, molecular mechanisms, and public health implications. Toxins.

[156] Chu M.C., Kreiswirth B.N., Pattee P.A., Novick R.P., Melish M.E., James J.F. (1988). Association of toxic shock toxin-1 determinant with a heterologous insertion at multiple loci in the *Staphylococcus aureus* chromosome. Infect. Immun..

[157] Mlynarczyk A., Mlynarczyk G., Jeljaszewicz J. (1998). The genome of *Staphylococcus aureus*: A review. Zentralbl Bakteriol..

[158] Wan T.W., Mori H., Hung W.C., Teng L.J., Yamamoto T. (2022). The phenomenon of staphylococcal interbacterial aggregate and net structures (SIAN) in community-associated methicillin-resistant *Staphylococcus aureus* CA-MRSA/J. J. Infect. Chemother..

[159] Subedi A., Ubeda C., Adhikari R.P., Penadés J.R., Novick R.P. (2007). Sequence analysis reveals genetic exchanges and intraspecific spread of SaPI2, a pathogenicity island involved in menstrual toxic shock. Microbiology.

[160] Yamaguchi T., Hayashi T., Takami H., Nakasone K., Ohnishi M., Nakayama K., Yamada S., Komatsuzawa H., Sugai M. (2000). Phage conversion of exfoliative toxin A production in *Staphylococcus aureus*. Mol. Microbiol..

[161] Yamaguchi T., Hayashi T., Takami H., Ohnishi M., Murata T., Nakayama K., Asakawa K., Ohara M., Komatsuzawa H., Sugai M. (2001). Complete nucleotide sequence of a *Staphylococcus aureus* exfoliative toxin B plasmid and identification of a novel ADP-ribosyltransferase, EDIN-C. Infect. Immun..

[162] Khokhlova O.E., Hung W.C., Wan T.W., Iwao Y., Takano T., Higuchi W., Yachenko S.V., Teplyakova O.V., Kamshilova V.V., Kotlovsky Y.V. (2015). Healthcare- and community-associated methicillin-resistant *Staphylococcus aureus* (MRSA) and fatal pneumonia with pediatric deaths in Krasnoyarsk, Siberian Russia: Unique MRSA’s multiple virulence factors, genome, and stepwise evolution. PLoS ONE.

[163] Amagai M., Matsuyoshi N., Wang Z.H., Andl C., Stanley J.R. (2000). Toxin in bullous impetigo and staphylococcal scalded-skin syndrome targets desmoglein 1. Nat. Med..

[164] Azarian T., Cella E., Baines S.L., Shumaker M.J., Samel C., Jubair M., Pegues D.A., David M.Z. (2021). Genomic epidemiology and global population structure of exfoliative toxin A-producing *Staphylococcus aureus* strains associated with staphylococcal scalded skin syndrome. Front. Microbiol..

[165] Bukowski M., Wladyka B., Dubin G. (2010). Exfoliative toxins of *Staphylococcus aureus*. Toxins.

[166] Vath G.M., Earhart C.A., Rago J.V., Kim M.H., Bohach G.A., Schlievert P.M., Ohlendorf G.H. (1997). The structure of the superantigen exfoliative toxin A suggests a novel regulation as a serine protease. Biochemistry.

[167] Ladhani S. (2001). Recent developments in staphylococcal scalded skin syndrome. Clin. Microbiol. Infect..

[168] Cribier B., Piemont Y., Grosshans E. (1994). Staphylococcal scalded skin syndrome in adults: A clinical review illustrated with a new case. J. Am. Acad. Dermatol..

[169] Supersac G., Prevost G., Piemont Y. (1993). Sequencing of leucocidin R from *Staphylococcus aureus* P83 suggests that staphylococcal leucocidins and gamma-hemolysin are members of a single, two-component family of toxins. Infect. Immun..

[170] Rahman A., Izaki K., Kamio Y. (1993). Gamma-hemolysin genes in the same family with *lukF* and *lukS* genes in methicillin resistant *Staphylococcus aureus*. Biosci. Biotechnol. Biochem..

[171] Dumitrescu O., Tristan A., Meugnier H., Bes M., Gouy M., Etienne J., Lina G., Vandenesch F. (2008). Polymorphism of the *Staphylococcus aureus* Panton-Valentine leukocidin genes and its possible link with the fitness of community-associated methicillin-resistant *S*. aureus. J. Infect. Dis..

[172] Gravet A., Colin D.A., Keller D., Girardot R., Monteil H., Prévost G. (1998). Characterization of a novel structural member, LukE-LukD, of the bi-component staphylococcal leucotoxins family. FEBS Lett..

[173] Lina G., Bohach G.A., Nair S.P., Hiramatsu K., Jouvin-Marche E., Mariuzza R. (2004). International Nomenclature Committee for Staphylococcal Superantigens. Standard nomenclature for the superantigens expressed by Staphylococcus. J. Infect. Dis..

[174] Barg N.L., Harris T., Crossley K.B., Archer G.L. (1997). Toxin-Mediąted Syndromes. The Staphylococci in Human Disease.

[175] Marr J.C., Lyon J.D., Roberson J.R., Lupher M., Davis W.C., Bohach G.A. (1993). Characterization of novel type C staphylococcal enterotoxins: Biological and evolutionary implications. Infect. Immun..

[176] Perez-Novo C.A., Zhang Y., Denil S., Trooskens G., De Meyer T., Van Criekinge W., Van Cauwenberge P., Zhang L., Bachert C. (2013). Staphylococcal enterotoxin B influences the DNA methylation pattern in nasal polyp tissue: A preliminary study. Allergy Asthma Clin. Immunol..

[177] Omoe K., Hu D.L., Ono H.K., Shimizu S., Takahashi-Omoe H., Nakane A., Uchiyama T., Shinagawa K., Imanishi K. (2013). Emetic potentials of newly identified staphylococcal enterotoxin-like toxins. Infect. Immun..

[178] Thomas D.Y., Jarraud S., Lemercier B., Cozon G., Echasserieau K., Etienne J., Gougeon M.-L., Lina G., Vandenesch F. (2006). Staphylococcal enterotoxin-like toxins U2 and V, two new staphylococcal superantigens arising from recombination within the enterotoxin gene cluster. Infect. Immun..

[179] Ono H.K., Sato’o Y., Narita K., Naito I., Hirose S., Hisatsune J., Asano K., Hu D.L., Omoe K., Sugai M. (2015). Identification and characterization of a novel staphylococcal emetic toxin. Appl. Environ. Microbiol..

[180] Argudin M.Á., Mendoza M.C., Rodicio M.R. (2010). Food poisoning and *Staphylococcus aureus* enterotoxins. Toxins.

[181] Miller C.R., Dey S., Smolenski P.D., Kulkarni P.S., Monk J.M., Szubin R., Sakoulas G., Berti A.D. (2020). Distinct subpopulations of intravalvular methicillin-resistant *Staphylococcus aureus* with variable susceptibility to daptomycin in tricuspid valve endocarditis. Antimicrob. Agents Chemother..

[182] Omoe K., Ishikawa M., Shimoda Y., Hu D.L., Ueda S., Shinagawa K. (2002). Detection of *seg*, *seh*, and *sei* genes in *Staphylococcus aureus* isolates and determination of the enterotoxin productivities of *S. aureus* isolates harboring *seg*, *seh*, or *sei* genes. J. Clin. Microbiol..

[183] Yarwood J.M., McCormick J.K., Paustian M.L., Orwin P.M., Kapur V., Schlievert P.M. (2002). Characterization and expression analysis of *Staphylococcus aureus* pathogenicity island 3. Implications for the evolution of staphylococcal pathogenicity islands. J. Biol. Chem..

[184] Sato’o Y., Omoe K., Ono H.K., Nakane A., Hu D.L. (2013). A novel comprehensive analysis method for *Staphylococcus aureus* pathogenicity islands. Microbiol. Immunol..

[185] Fanelli F., Chieffi D., Cho G.S., Schubert J., Mekhloufi O.A., Bania J., Franz C.M.A.P., Fusco V. (2022). First genome-based characterisation and staphylococcal enterotoxin production ability of methicillin-susceptible and methicillin-resistant *Staphylococcus aureus* strains isolated from ready-to-eat foods in Algiers (Algeria). Toxins.

[186] Suzuki Y., Omoe K., Hu D.L., Sato’o Y., Ono H.K., Monma C., Arai T., Konishi N., Kato R., Hirai A. (2014). Molecular epidemiological characterization of *Staphylococcus aureus* isolates originating from food poisoning outbreaks that occurred in Tokyo, Japan. Microbiol. Immunol..

[187] Steinig E.J., Andersson P., Harris S.R., Sarovich D.S., Manoharan A., Coupland P., Holden M.T., Parkhill J., Bentley S.D., Robinson D.A. (2015). Single-molecule sequencing reveals the molecular basis of multidrug-resistance in ST772 methicillin-resistant *Staphylococcus aureus*. BMC Genom..

[188] Li Z., Stevens D.L., Hamilton S.M., Parimon T., Ma Y., Kearns A.M., Ellis R.W., Bryant A.E. (2011). Fatal *S. aureus* hemorrhagic pneumonia: Genetic analysis of a unique clinical isolate producing both PVL and TSST-1. PLoS ONE.

[189] Bayles K.W., Iandolo J.J. (1989). Genetic and molecular analyses of the gene encoding staphylococcal enterotoxin D. J. Bacteriol..

[190] Sato’o Y., Omoe K., Aikawa Y., Kano M., Ono H.K., Hu D.L., Nakane A., Sugai M. (2021). Investigation of *Staphylococcus aureus* positive for Staphylococcal enterotoxin S and T genes. J. Vet. Med. Sci..

[191] Chieffi D., Fanelli F., Cho G.S., Schubert J., Blaiotta G., Franz C.M.A.P., Bania J., Fusco V. (2020). Novel insights into the enterotoxigenic potential and genomic background of *Staphylococcus aureus* isolated from raw milk. Food Microbiol..

[192] Ono H.K., Omoe K., Imanishi K., Iwakabe Y., Hu D.L., Kato H., Saito N., Nakane A., Uchiyama T., Shinagawa K. (2008). Identification and characterization of two novel staphylococcal enterotoxins, types S and T. Infect. Immun..

[193] Aung M.S., Urushibara N., Kawaguchiya M., Ito M., Habadera S., Kobayashi N. (2020). Prevalence and genetic diversity of staphylococcal enterotoxin (-like) genes *sey*, *selw*, *selx*, *selz*, *sel26* and *sel27* in community-acquired methicillin-resistant *Staphylococcus aureus*. Toxins.

[194] Gawlik D., Ruppelt-Lorz A., Müller E., Reißig A., Hotzel H., Braun S.D., Söderquist B., Ziegler-Cordts A., Stein C., Pletz M.W. (2020). Molecular investigations on a chimeric strain of *Staphylococcus aureus* sequence type 80. PLoS ONE.

[195] Sanchez-Morgado J.M. (2023). The role of environmental enrichment in the aetiopathogenesis of *Staphylococcus aureus* facial, periorbital and retroorbital abscesses in mice. Lab. Anim..

[196] Zhang D.F., Yang X.Y., Zhang J., Qin X., Huang X., Cui Y., Zhou M., Shi C., French N.P., Shi X. (2018). Identification and characterization of two novel superantigens among *Staphylococcus aureus* complex. Int. J. Med. Microbiol..

[197] Blicharz L., Żochowski M., Szymanek-Majchrzak K., Czuwara J., Goldust M., Skowroński K., Młynarczyk G., Olszewska M., Samochocki Z., Rudnicka L. (2022). Enterotoxin gene cluster and *selX* are associated with atopic dermatitis severity-a cross-sectional molecular study of *Staphylococcus aureus* superantigens. Cells.

[198] Ohnishi Y., Okino N., Ito M., Imayama S. (1999). Ceramidase activity in bacterial skin flora as a possible cause of ceramide deficiency in atopic dermatitis. Clin. Diagn. Lab. Immunol..

[199] Pickering A.C., Yebra G., Gong X., Goncheva M.I., Wee B.A., MacFadyen A.C., Muehlbauer L.F., Alves J., Cartwright R.A., Paterson G.K. (2021). Evolutionary and functional analysis of coagulase positivity among the Staphylococci. mSphere.

[200] Courjon J., Munro P., Benito Y., Visvikis O., Bouchiat C., Boyer L., Doye A., Lepidi H., Ghigo E., Lavigne J.P. (2015). EDIN-B promotes the translocation of *Staphylococcus aureus* to the bloodstream in the course of pneumonia. Toxins.

[201] Phonimdaeng P., O’Reilly M., Nowlan P., Bramley A.J., Foster T.J. (1990). The coagulase of *Staphylococcus aureus* 8325-4. Sequence analysis and virulence of site-specific coagulase-deficient mutants. Mol. Microbiol..

[202] Sawai T., Tomono K., Yanagihara K., Yamamoto Y., Kaku M., Hirakata Y., Koga H., Tashiro T., Kohno S. (1997). Role of coagulase in a murine model of hematogenous pulmonary infection induced by intravenous injection of *Staphylococcus aureus* enmeshed in agar beads. Infect. Immun..

[203] Kaida S., Miyata T., Yoshizawa Y., Igarashi H., Iwanaga S. (1989). Nucleotide and deduced amino acid sequences of staphylocoagulase gene from *Staphylococcus aureus* strain 213. Nucleic Acids Res..

[204] Kaida S., Miyata T., Yoshizawa Y., Kawabata S., Morita T., Igarashi H., Iwanaga S. (1987). Nucleotide sequence of the staphylocoagulase gene: Its unique COOH-terminal 8 tandem repeats. J. Biochem..

[205] Yu J., Jiang F., Zhang F., Hamushan M., Du J., Mao Y., Wang Q., Han P., Tang J., Shen H. (2021). Thermonucleases contribute to *Staphylococcus aureus* biofilm formation in implant-associated infections-a redundant and complementary story. Front. Microbiol..

[206] Li X., Maaß S., Ferrero-Bordera B., Zhang Z., Wang M., Sietsema E., Liu L., Divinagracia M., van Dijl J.M., Buist G. (2025). The secreted proteases *aur*, *scpA*, *sspA and sspB* suppress the virulence of *Staphylococcus aureus* USA300 by shaping the extracellular proteome. Virulence.

[207] Kline S.N., Saito Y., Archer N.K. (2024). *Staphylococcus aureus* proteases: Orchestrators of skin inflammation. DNA Cell Biol..

[208] Inoue S., Sugai M., Murooka Y., Paik S.Y., Hong Y.M., Ohgai H., Suginaka H. (1991). Molecular cloning and sequencing of the epidermal cell differentiation inhibitor gene from *Staphylococcus aureus*. Biochem. Biophys. Res. Commun..

[209] Haley K.P., Skaar E.P. (2012). A battle for iron: Host sequestration and *Staphylococcus aureus* acquisition. Microbes Infect..

[210] Xiong A., Singh V.K., Cabrera G., Jayaswal R.K. (2000). Molecular characterization of the ferric-uptake regulator, fur, from *Staphylococcus aureus*. Microbiology.

[211] Torres V.J., Attia A.S., Mason W.J., Hood M.I., Corbin B.D., Beasley F.C., Anderson K.L., Stauff D.L., McDonald W.H., Zimmerman L.J. (2010). *Staphylococcus aureus* fur regulates the expression of virulence factors that contribute to the pathogenesis of pneumonia. Infect. Immun..

[212] Grigg J.C., Ukpabi G., Gaudin C.F., Murphy M.E. (2010). Structural biology of heme binding in the *Staphylococcus aureus* Isd system. J. Inorg. Biochem..

[213] Mazmanian S.K., Liu G., Jensen E.R., Lenoy E., Schneewind O. (2000). *Staphylococcus aureus* sortase mutants defective in the display of surface proteins and in the pathogenesis of animal infections. Proc. Natl. Acad. Sci. USA.

[214] Pilpa R.M., Fadeev E.A., Villareal V.A., Wong M.L., Phillips M., Clubb R.T. (2006). Solution structure of the NEAT (NEAr Transporter) domain from IsdH/HarA: The human hemoglobin receptor in *Staphylococcus aureus*. J. Mol. Biol..

[215] Grigg J.C., Vermeiren C.L., Heinrichs D.E., Murphy M.E. (2007). Haem recognition by a *Staphylococcus aureus* NEAT domain. Mol. Microbiol..

[216] Liu M., Tanaka W.N., Zhu H., Xie G., Dooley D.M., Lei B. (2008). Direct hemin transfer from IsdA to IsdC in the iron-regulated surface determinant (Isd) heme acquisition system of *Staphylococcus aureus*. J. Biol. Chem..

[217] Muryoi N., Tiedemann M.T., Pluym M., Cheung J., Heinrichs D.E., Stillman M.J. (2008). Demonstration of the iron-regulated surface determinant (Isd) heme transfer pathway in *Staphylococcus aureus*. J. Biol. Chem..

[218] Skaar E.P., Gaspar A.H., Schneewind O. (2004). IsdG and IsdI, heme-degrading enzymes in the cytoplasm of *Staphylococcus aureus*. J. Biol. Chem..

[219] Gianquinto E., Moscetti I., De Bei O., Campanini B., Marchetti M., Luque F.J., Cannistraro S., Ronda L., Bizzarri A.R., Spyrakis F. (2019). Interaction of human hemoglobin and semi-hemoglobins with the *Staphylococcus aureus* hemophore IsdB: A kinetic and mechanistic insight. Sci. Rep..

[220] De Bei O., Marchetti M., Guglielmo S., Gianquinto E., Spyrakis F., Campanini B., Bettati S., Levantino M., Ronda L. (2025). Time-resolved X-ray solution scattering unveils the events leading to hemoglobin heme capture by staphylococcal IsdB. Nat. Commun..

[221] Pishchany G., McCoy A.L., Torres V.J., Krause J.C., Crowe J.E., Fabry M.E., Skaar E.P. (2010). Specificity for human hemoglobin enhances *Staphylococcus aureus* infection. Cell Host Microbe.

[222] Wright J.A., Nair S.P. (2012). The lipoprotein components of the Isd and Hts transport systems are dispensable for acquisition of heme by *Staphylococcus aureus*. FEMS Microbiol. Lett..

[223] Conroy B.S., Grigg J.C., Kolesnikov M., Morales L.D., Murphy M.E.P. (2019). *Staphylococcus aureus* heme and siderophore-iron acquisition pathways. Biometals.

[224] Friedman D.B., Stauff D.L., Pishchany G., Whitwell C.W., Torres V.J., Skaar E.P. (2006). *Staphylococcus aureus* redirects central metabolism to increase iron availability. PLoS Pathog..

[225] Lindsay J.A., Riley T.V. (1994). Staphylococcal iron requirements, siderophore production, and iron-regulated protein expression. Infect. Immun..

[226] Beasley F.C., Heinrichs D.E. (2010). Siderophore-mediated iron acquisition in the staphylococci. J. Inorg. Biochem..

[227] Beasley F.C., Vinés E.D., Grigg J.C., Zheng Q., Liu S., Lajoie G.A., Murphy M.E., Heinrichs D.E. (2009). Characterization of staphyloferrin A biosynthetic and transport mutants in *Staphylococcus aureus*. Mol. Microbiol..

[228] Skaar E.P., Humayun M., Bae T., DeBord K.L., Schneewind O. (2004). Iron-source preference of *Staphylococcus aureus* infections. Science.

[229] Mayville P., Ji G., Beavis R., Yang H., Goger M., Novick R.P., Muir T.W. (1999). Structure-activity analysis of synthetic autoinducing thiolactone peptides from *Staphylococcus aureus* responsible for virulence. Proc. Natl. Acad. Sci. USA.

[230] Szymanek K., Młynarczyk A., Młynarczyk G. (2009). Regulatory systems of gene expresion in *Staphylococcus aureus*. Post. Mikrobiol..

[231] Patel H., Rawat S. (2023). A genetic regulatory see-saw of biofilm and virulence in MRSA pathogenesis. Front. Microbiol..

[232] Novick R.P., Ross H.F., Projan S.J., Kornblum J., Kreiswirth B., Moghazeh S. (1993). Synthesis of staphylococcal virulence factors is controlled by a regulatory RNA molecule. EMBO J..

[233] Dunman P.M., Murphy E., Haney S., Palacios D., Tucker-Kellogg G., Wu S., Brown E.L., Zagursky R.J., Shlaes D., Projan S.J. (2001). Transcription profiling-based identification of *Staphylococcus aureus* genes regulated by the *agr* and/or *sarA* loci. J. Bacteriol..

[234] Jenul C., Horswill A.R. (2019). Regulation of *Staphylococcus aureus* virulence. Microbiol. Spectr..

[235] Butrico C.E., Cassat J.E. (2020). Quorum sensing and toxin production in *Staphylococcus aureus* osteomyelitis: Pathogenesis and paradox. Toxins.

[236] Paharik A.E., Parlet C.P., Chung N., Todd D.A., Rodriguez E.I., Van Dyke M.J., Cech N.B., Horswill A.R. (2017). Coagulase-negative staphylococcal strain prevents *Staphylococcus aureus* colonization and skin infection by blocking quorum sensing. Cell Host Microbe.

[237] Wardenburg J.B., Patel R.J., Schneewind O. (2007). Surface proteins and exotoxins are required for the pathogenesis of *Staphylococcus aureus* pneumonia. Infect. Immun..

[238] Novick R.P., Geisinger E. (2008). Quorum sensing in staphylococci. Annu. Rev. Genet..

[239] Cheung A., Eberhardt K., Chung E., Yeaman M., Sullam P., Ramos M., Bayer A. (1994). Diminished virulence of a *sar-/agr*-mutant of *Staphylococcus aureus* in the rabbit model of endocarditis. J. Clin. Investig..

[240] Crosby H.A., Tiwari N., Kwiecinski J.M., Xu Z., Dykstra A., Jenul C., Fuentes E.J., Horswill A.R. (2020). The *Staphylococcus aureus* ArlRS two-component system regulates virulence factor expression through MgrA. Mol. Microbiol..

[241] Fournier B., Klier A., Rapoport G. (2001). The two-component system ArlS–ArlR is a regulator of virulence gene expression in *Staphylococcus aureus*. Mol. Microbiol..

[242] Walker J.N., Crosby H.A., Spaulding A.R., Salgado-Pabón W., Malone C.L., Rosenthal C.B., Schlievert P.M., Boyd J.M., Horswill A.R. (2013). The *Staphylococcus aureus* ArlRS two-component system is a novel regulator of agglutination and pathogenesis. PLoS Pathog..

[243] Kwiecinski J.M., Kratofil R.M., Parlet C.P., Surewaard B.G.J., Kubes P., Horswill A.R. (2021). *Staphylococcus aureus* uses the ArlRS and MgrA cascade to regulate immune evasion during skin infection. Cell Rep..

[244] Liang X., Yu C., Sun J., Liu H., Landwehr C., Holmes D., Ji Y. (2006). Inactivation of a two-component signal transduction system, SaeRS, eliminates adherence and attenuates virulence of *Staphylococcus aureus*. Infect. Immun..

[245] Olson M.E., Nygaard T.K., Ackermann L., Watkins R.L., Zurek O.W., Pallister K.B., Griffith S., Kiedrowski M.R., Flack C.E., Kavanaugh J.S. (2013). *Staphylococcus aureus* nuclease is an SaeRS-dependent virulence factor. Infect. Immun..

[246] Baroja M.L., Herfst C.A., Kasper K.J., Xu S.X., Gillett D.A., Li J., Reid G., McCormick J.K. (2016). The SaeRS two-component system is a direct and dominant transcriptional activator of toxic shock syndrome toxin 1 in *Staphylococcus aureus*. J. Bacteriol..

[247] Novick R.P., Jiang D. (2003). The staphylococcal saeRS system coordinates environmental signals with agr quorum sensing. Microbiology.

[248] Yarwood J.M., McCormick J.K., Schlievert P.M. (2001). Identification of a novel two-component regulatory system that acts in global regulation of virulence factors of *Staphylococcus aureus*. J. Bacteriol..

[249] Ulrich M., Bastian M., Cramton S.E., Ziegler K., Pragman A.A., Bragonzi A., Memmi G., Wolz C., Schlievert P.M., Cheung A. (2007). The staphylococcal respiratory response regulator SrrAB induces *ica* gene transcription and polysaccharide intercellular adhesin expression, protecting *Staphylococcus aureus* from neutrophil killing under anaerobic growth conditions. Mol. Microbiol..

[250] Cameron D.R., Jiang J.H., Kostoulias X., Foxwell D.J., Peleg A.Y. (2016). Vancomycin susceptibility in methicillin-resistant *Staphylococcus aureus* is mediated by YycHI activation of the WalRK essential two-component regulatory system. Sci. Rep..

[251] Dubrac S., Msadek T. (2004). Identification of genes controlled by the essential YycG/YycF two-component system of *Staphylococcus aureus*. J. Bacteriol..

[252] Wu S., Zhang J., Peng Q., Liu Y., Lei L., Zhang H. (2021). The role of *Staphylococcus aureus* YycFG in gene regulation, biofilm organization and drug resistance. Antibiotics.

[253] Beltrame C.O., Côrtes M.F., Bonelli R.R., Côrrea A.B., Botelho A.M., Américo M.A., Fracalanzza S.E., Figueiredo A.M. (2015). Inactivation of the autolysis-related genes *lrgB* and *yycI* in *Staphylococcus aureus* increases cell lysis-dependent eDNA release and enhances biofilm development in vitro and in vivo. PLoS ONE.

[254] Kraus D., Herbert S., Kristian S.A., Khosravi A., Nizet V., Götz F., Peschel A. (2008). The GraRS regulatory system controls *Staphylococcus aureus* susceptibility to antimicrobial host defenses. BMC Microbiol..

[255] Gardete S., Kim C., Hartmann B.M., Mwangi M., Roux C.M., Dunman P.M., Chambers H.F., Tomasz A. (2012). Genetic pathway in acquisition and loss of vancomycin resistance in a methicillin resistant *Staphylococcus aureus* (MRSA) strain of clonal type USA300. PLoS Pathog..

[256] Freeman Z.N., Dorus S., Waterfield N.R. (2013). The KdpD/KdpE two-component system: Integrating K^+^ homeostasis and virulence. PLoS Pathog..

[257] Parsons J.B., Broussard T.C., Bose J.L., Rosch J.W., Jackson P., Subramanian C., Rock C.O. (2014). Identification of a two-component fatty acid kinase responsible for host fatty acid incorporation by *Staphylococcus aureus*. Proc. Natl. Acad. Sci. USA.

[258] Ju Y., An Q., Zhang Y., Sun K., Bai L., Luo Y. (2021). Recent advances in Clp protease modulation to address virulence, resistance and persistence of MRSA infection. Drug Discov. Today.

[259] Tamber S., Reyes D., Donegan N.P., Schwartzman J.D., Cheung A.L., Memmi G. (2010). The staphylococcus-specific gene *rsr* represses *agr* and virulence in *Staphylococcus aureus*. Infect. Immun..

[260] Seidl K., Stucki M., Ruegg M., Goerke C., Wolz C., Harris L., Berger-Bächi B., Bischoff M. (2006). *Staphylococcus aureus* CcpA affects virulence determinant production and antibiotic resistance. Antimicrob. Agents Chemother..

[261] Fang B., Liu B., Sun B. (2020). Transcriptional regulation of virulence factors Hla and phenol-soluble modulins α by AraC-type regulator Rbf in *Staphylococcus aureus*. Int. J. Med. Microbiol..

[262] Jobson M.-E., Tomlinson B.R., Mustor E.M., Felton E.A., Weiss A., Caswell C.C., Shaw L.N. (2025). SSR42 is a novel regulator of cytolytic activity in *Staphylococcus aureus*. mBio.

[263] McNamara P.J., Milligan-Monroe K.C., Khalili S., Proctor R.A. (2000). Identification, cloning, and initial characterization of rot, a locus encoding a regulator of virulence factor expression in *Staphylococcus aureus*. J. Bacteriol..

[264] Said-Salim B., Dunman P., McAleese F., Macapagal D., Murphy E., McNamara P., Arvidson S., Foster T., Projan S., Kreiswirth B. (2003). Global regulation of *Staphylococcus aureus* genes by rot. J. Bacteriol..

[265] Crosby H.A., Schlievert P.M., Merriman J.A., King J.M., Salgado-Pabón W., Horswill A.R. (2016). The *Staphylococcus aureus* global regulator MgrA modulates clumping and virulence by controlling surface protein expression. PLoS Pathog..

[266] Entenza J.-M., Moreillon P., Senn M.M., Kormanec J., Dunman P.M., Berger-Bächi B., Projan S., Bischoff M. (2005). Role of σB in the expression of *Staphylococcus aureus* cell wall adhesins ClfA and FnbA and contribution to infectivity in a rat model of experimental endocarditis. Infect. Immun..

[267] Tuchscherr L., Bischoff M., Lattar S.M., Noto Llana M., Pförtner H., Niemann S., Geraci J., Van de Vyver H., Fraunholz M.J., Cheung A.L. (2015). Sigma factor SigB is crucial to mediate *Staphylococcus aureus* adaptation during chronic infections. PLoS Pathog..

[268] Augagneur Y., King A.N., Germain-Amiot N., Sassi M., Fitzgerald J.W., Sahukhal G.S., Elasri M.O., Felden B., Brinsmade S.R. (2020). Analysis of the CodY RNome reveals RsaD as a stress-responsive riboregulator of overflow metabolism in *Staphylococcus aureus*. Mol. Microbiol..

[269] Wittekind M.A., Frey A., Bonsall A.E., Briaud P., Keogh R.A., Wiemels R.E., Shaw L.N., Carroll R.K. (2022). The novel protein ScrA acts through the SaeRS two-component system to regulate virulence gene expression in *Staphylococcus aureus*. Mol. Microbiol..

[270] Tegmark K., Karlsson A., Arvidson S. (2000). Identification and characterization of SarH1, a new global regulator of virulence gene expression in *Staphylococcus aureus*. Mol. Microbiol..

[271] Cheung A.L., Bayer A.S., Zhang G., Gresham H., Xiong Y.-Q. (2004). Regulation of virulence determinants in vitro and in vivo in *Staphylococcus aureus*. FEMS Immunol. Med. Microbiol..

[272] Zielinska A.K., Beenken K.E., Mrak L.N., Spencer H.J., Post G.R., Skinner R.A., Tackett A.J., Horswill A.R., Smeltzer M.S. (2012). *sarA*-mediated repression of protease production plays a key role in the pathogenesis of *Staphylococcus aureus* USA 300 isolates. Mol. Microbiol..

[273] Manna A., Cheung A.L. (2001). Characterization of *sarR*, a modulator of sar expression in *Staphylococcus aureus*. Infect. Immun..

[274] Schmidt K.A., Manna A.C., Gill S., Cheung A.L. (2001). SarT, a repressor of alpha-hemolysin in *Staphylococcus aureus*. Infect. Immun..

[275] Bischoff M., Wonnenberg B., Nippe N., Nyffenegger-Jann N.J., Voss M., Beisswenger C., Sunderkotter C., Molle V., Dinh Q.T., Lammert F. (2017). CcpA affects infectivity of *Staphylococcus aureus* in a hyperglycemic environment. Front. Cell Infect. Microbiol..

[276] Manna A.C., Ingavale S.S., Maloney M., van Wamel W., Cheung A.L. (2004). Identification of *sarV* (SA2062), a new transcriptional regulator, is repressed by SarA and MgrA (SA0641) and involved in the regulation of autolysis in *Staphylococcus aureus*. J. Bacteriol..

[277] Klaui A.J., Boss R., Graber H.U. (2019). Characterization and comparative analysis of the *Staphylococcus aureus* genomic island vSabeta: An in silico approach. J. Bacteriol..

[278] Collery M.M., Smyth D.S., Tumilty J.J.G., Twohig J.M., Smyth C.J. (2009). Associations between enterotoxin gene cluster types egc1, egc2 and egc3, agr types, enterotoxin and enterotoxin-like gene profiles, and molecular typing characteristics of human nasal carriage and animal isolates of *Staphylococcus aureus*. J. Med. Microbiol..

[279] Letertre C., Perelle S., Dilasser F., Fach P. (2003). Identification of a new putative enterotoxin SEU encoded by the egc cluster of *Staphylococcus aureus*. J. Appl. Microbiol..

[280] Baba T., Takeuchi F., Kuroda M., Yuzawa H., Aoki K., Oguchi A., Nagai Y., Iwama N., Asano K., Naimi T. (2002). Genome and virulence determinants of high virulence community-acquired MRSA. Lancet.

[281] Baba T., Bae T., Schneewind O., Takeuchi F., Hiramatsu K. (2008). Genome sequence of *Staphylococcus aureus* strain Newman and comparative analysis of staphylococcal genomes: Polymorphism and evolution of two major pathogenicity islands. J. Bacteriol..

[282] Alibayov B., Baba-Moussa L., Sina H., Zdeňková K., Demnerová K. (2014). *Staphylococcus aureus* mobile genetic elements. Mol. Biol. Rep..

[283] Jarraud S., Peyrat M.A., Lim A., Tristan A., Bes M., Mougel C., Etienne J., Vandenesch F., Bonneville M., Lina G. (2001). egc, a highly prevalent operon of enterotoxin gene, forms a putative nursery of superantigens in *Staphylococcus aureus*. J. Immunol..

[284] Novick R.P., Subedi A. (2007). The SaPIs: Mobile pathogenicity islands of *Staphylococcus*. Chem. Immunol. Allergy.

[285] Jin Y., Zhou W., Ge Q., Shen P., Xiao Y. (2024). Epidemiology and clinical features of skin and soft tissue infections caused by PVL-positive and PVL-negative methicillin-resistant *Staphylococcus aureus* isolates in inpatients in China: A single-center retrospective 7-year study. Emerg. Microbes Infect..

[286] Nakamura Y., Takahashi H., Takaya A., Inoue Y., Katayama Y., Kusuya Y., Shoji T., Takada S., Nakagawa S., Oguma R. (2020). *Staphylococcus* Agr virulence is critical for epidermal colonization and associates with atopic dermatitis development. Sci. Transl. Med..

[287] Travers J.B., Kozman A., Mousdicas N., Saha C., Landis M., Al-Hassani M., Yao W., Yao Y., Hyatt A.M., Sheehan M.P. (2010). Infected atopic dermatitis lesions contain pharmacologic amounts of lipoteichoic acid. J. Allergy Clin. Immunol..

[288] Blicharz L., Michalak M., Szymanek-Majchrzak K., Młynarczyk G., Skowroński K., Rudnicka L., Samochocki Z. (2021). The propensity to form biofilm in vitro by *Staphylococcus aureus* strains Isolated from the anterior nares of patients with atopic dermatitis: Clinical associations. Dermatology.

[289] Blicharz L., Szymanek-Majchrzak K., Młynarczyk G., Czuwara J., Waśkiel-Burnat A., Goldust M., Samochocki Z., Rudnicka L. (2023). Multilocus-sequence typing reveals clonality of *Staphylococcus aureus* in atopic dermatitis. Clin. Exp. Dermatol..

[290] Sroka-Tomaszewska J., Trzeciak M. (2021). Molecular mechanisms of atopic dermatitis pathogenesis. Int. J. Mol. Sci..

[291] Simpson E.L., Schlievert P.M., Yoshida T., Lussier S., Boguniewicz M., Hata T., Fuxench Z., De Benedetto A., Ong P.Y., Ko J. (2023). Rapid reduction in *Staphylococcus aureus* in atopic dermatitis subjects following dupilumab treatment. J. Allergy Clin. Immunol..

[292] Vadivel C.K., Willerslev-Olsen A., Namini M.R.J., Zeng Z., Yan L., Danielsen M., Gluud M., Pallesen E.M.H., Wojewoda K., Osmancevic A. (2024). *Staphylococcus aureus* induces drug resistance in cancer T cells in Sézary syndrome. Blood.

[293] Liu Y., Wu X., Song P., Liu L., Zhong X., He Q., Zhang Z. (2024). Increased *S. aureus* colonization and reduced antimicrobial peptide expression in erythrodermic psoriasis. Int. Immunopharmacol..

[294] Cucu C.I., Giurcăneanu C., Poenaru E., Popa L.G., Popa M.I., Chifiriuc M.C., Lazăr V., Holban A.M., Gheorghe-Barbu I., Muntean A.A. (2025). Phenotypic and genotypic bacterial virulence and resistance profiles in hidradenitis suppurativa. Int. J. Mol. Sci..

[295] Stergianou D., Tzanetakou V., Argyropoulou M., Kanni T., Bagos P.G., Giamarellos-Bourboulis E.J. (2021). *Staphylococcus aureus* carriage in hidradenitis suppurativa: Impact on response to adalimumab. Dermatology.

